# Male cooperation for breeding opportunities contributes to the evolution of multilevel societies

**DOI:** 10.1098/rspb.2017.1480

**Published:** 2017-09-27

**Authors:** Xiao-Guang Qi, Kang Huang, Gu Fang, Cyril C. Grueter, Derek W. Dunn, Yu-Li Li, Weihong Ji, Xiao-Yan Wang, Rong-Tao Wang, Paul A. Garber, Bao-Guo Li

**Affiliations:** 1 College of Life Sciences, Northwest University, Xi'an 710069, China; 2 Shaanxi Key Laboratory for Animal Conservation, Northwest University, Xi'an 710069, China; 3 Anthropology Department, University of Illinois, Urbana, IL 61801, USA; 4 School of Human Sciences, The University of Western Australia, Perth, Western Australia 6009, Australia; 5 Institute of Natural and Mathematical Sciences, Massey University, Albany, Auckland, New Zealand

**Keywords:** all-male group, multilevel society, kinship, social network analysis, primates social evolution

## Abstract

A small number of primate species including snub-nosed monkeys (colobines), geladas (papionins) and humans live in multilevel societies (MLSs), in which multiple one-male polygamous units (OMUs) coexist to form a band, and non-breeding males associate in bachelor groups. Phylogenetic reconstructions indicate that the papionin MLS appears to have evolved through internal fissioning of large mixed-sex groups, whereas the colobine MLS evolved through the aggregation of small, isolated OMUs. However, how agonistic males maintain tolerance under intensive competition over limited breeding opportunities remains unclear. Using a combination of behavioural analysis, satellite telemetry and genetic data, we quantified the social network of males in a bachelor group of golden snub-nosed monkeys. The results show a strong effect of kinship on social bonds among bachelors. Their interactions ranged from cooperation to agonism, and were regulated by access to mating partners. We suggest that an ‘arms race’ between breeding males' collective defence against usurpation attempts by bachelor males and bachelor males' aggregative offence to obtain reproductive opportunities has selected for larger group size on both sides. The results provide insight into the role that kin selection plays in shaping inter-male cohesion which facilities the evolution of multilevel societies. These findings have implications for understanding human social evolution, as male–male bonds are a hallmark of small- and large-scale human societies.

## 1. Introduction

In many species of primates, including humans, affiliative bonding and alliances among resident males play an important role in determining the costs and benefits of social group living [1–4]. These social interactions vary among species from mainly solitary individuals that occasionally forage or mate together, to permanent and complex multilevel societies (MLSs) similar to those of humans [5]. Many non-human primate social systems are based around small family groups [6,7]. Each group occupies an exclusive territory and avoids others [8]. These groups may consist principally of a single, adult breeding male, multiple harem females and their immature offspring (one-male polygamous unit, OMU), such as in some species of leaf-eating langur and colobus (Colobinae) [9], or a single monogamous adult male and a single female, and their juvenile offspring, such as reported in some gibbon species [10,11]. By contrast, a small number of primates form an MLS consisting of several of these family units living in close coordination, forming a larger group of up to several hundreds of individuals [12].

Primate MLSs are rare; in addition to humans, they have been reported only in Asian colobines including snub-nosed monkeys (*Rhinopithecus* spp.) and proboscis monkeys (*Nasalis larvatus*), and in African papionins such as hamadryas (*Papio hamadryas*) and Guinea (*Papio papio*) baboons, as well as geladas (*Theropithecus gelada*) [13,14]. Primate MLSs usually consist of a breeding band (BB) that comprises several coexisting OMUs, and an all-male band (AMB or bachelor group) that shadows the BB. Together, the AMB and the BB form a herd [2]. The AMB consists of those young males waiting for reproductive opportunities and former OMU resident males whose harem has been taken over and are presently excluded from the BB [15]. Although the evolution of an MLS from small groups is likely to have required increased male–male tolerance and social coordination among different families, the specific selective mechanisms favouring MLS formation still remain unidentified [16]. Moreover, an increased understanding of the dynamics of male–male alliances in primate MLSs is important, especially because it has been argued that similar processes may have influenced social evolution in humans, owing to sociality and cooperation possibly forming in the same way in ancestral and modern hunter-gatherer humans [17–19].

Primate MLSs have been proposed to have evolved via two independent pathways. The first is via the fission of large *multi-male, multi-female* groups, as in African papionins. Owing to increased intra-sexual competition, there would be selection for enhanced sexually dimorphic traits in males, such as larger body size, to enable single males to monopolize multiple females [20,21]. This results in a few resident males maintaining exclusive access to several female mating partners, forming several OMUs nested within the group along with multiple subordinate males excluded from reproductive opportunities. This suggests that the MLS of species in some African papionins appears to have evolved through such a process associated with the internal fissioning of a single large multi-male, multi-female group into an MLS comprising a set of OMUs [12,16] with less competitive males being excluded and forming a small AMB [22].

The second evolutionary pathway to an MLS is via the fusion of multiple independent OMUs into a single large BB [2,23]. The ancestral social condition for Asian colobines is likely to be isolated OMUs, each of whose territory overlapped with solitary bachelor males or small all-male units (AMU) consisting of only a few bachelor males (e.g. [24]). It has been suggested that the snub-nosed monkey MLS evolved from an aggregation of several independent OMUs into a single cohesive BB [2,23], in response to selection favouring the collective action of breeding males from multiple OMUs to form defensive alliances against potential predators as well as to combat takeovers by bachelor males [23,25,26].

In response to challenges faced by the increased defensive capabilities of breeding male alliances, as well as predators [26,27], bachelor males could also have been selected to increase tolerance and transition from solitariness into a bonded group, thus permitting the formation of AMB inter-male alliances. Here, we assume that this may have resulted in the evolution of larger AMBs and larger BBs in response to an ‘arms race’ over access to reproductively active females. It is possible that over time, male alliances within both the BBs and AMBs would act to increase group size, resulting in the large snub-nosed monkey herds we see at present, especially under conditions of abundant and/or evenly distributed food resources.

To more fully understand the contradictory roles of reproductive competition and male alliances in the evolution of a snub-nosed monkey MLS, detailed information is required on the patterns of male tolerance and affiliation among bachelor males residing within the AMB, as well as the interaction dynamics between males of the AMB and the BB as they compete for reproductive opportunities. We used satellite telemetry, behavioural observations and genetic analysis to study the AMB in an MLS of the golden snub-nosed monkey (*Rhinopithecus roxellana*) in central China. We present, to our knowledge, the first quantitative study of the relative contribution of kinship, dominance rank and age on male social associations. These results help us to understand how male–male bonds are formed and how these alliances change in response to changes when reproductive opportunities arise and ultimately how male–male interactions contribute to the formation of the MLS.

Our results provide new evidences to support the fusion hypothesis for the evolution of primate MLSs, and suggest that the formation of kin-bonds among bachelor males offers a critical insight into the evolution of the *R. roxellana* MLS, as well as possibly in other primates including humans.

## 2. Methods

### (a) Study site and subject

This study was conducted on a wild troop (West Ridge troop, WRT) of *R. roxellana* inhabiting the Yuhuangmiao area within the Zhouzhi National Nature Reserve (ZNNR) of the Qinling Mountains, China. The WRT consists of the GNG-herd and the DJF-herd. Each herd contains a BB, formed of 7–13 OMUs, and an AMB, as well as some solitary males that move and forage independently. The two herds have partially overlapping home ranges, with seasonal fuse during a brief period each year. Individuals sometimes transfer between herds [2,28] (see the electronic supplementary material for details).

### (b) Behavioural analysis

#### (i) Affiliation and social network analysis

We used social network analysis (SNA) and Socprog v. 2.1 [29] to measure patterns of individual social association within the GNG-all-male band (GNG-AMB). Affiliation behaviour was classified as an ‘association’ to describe indirect/non-contact affiliation, and ‘interaction’ to describe direct and instantaneous behaviour from one individual to another. We used proximity whereby two or more individuals shared the same space at the same time to represent associations, and grooming to represent interactions.

Proximity data were collected using a 5 min interval scan sampling technique. The affiliation index was calculated based on the half-weight index (HWI) [30] to measure the strength of social associations between individuals within the AMB (see the electronic supplementary material for details).

Grooming data were collected using an all-occurrence sampling method, and the interactions between each individual within the AMB were measured with the directional affiliation index (DAI):
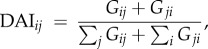
where *G_ij_* is the total number of times grooming was initiated by individual *i* to *j*. Sociograms to describe the patterns of individual association and interaction were constructed using Socprog v. 2.1 [31].

Individuals were identified based on their physical characteristics, radio-frequency identification tags or a tattoo placed on their lips. DNA fingerprinting was also used to identify individuals (see the electronic supplementary material for details).

#### (ii) Cliquishness and *COMMUNITY*

To estimate cliquishness or evidence of social networks composed of multiple individuals, we performed a hierarchical clustering analysis (HCA) of SNA. Each clique represented a subgroup or an all-male unit within the AMB.

To evaluate the stability of individual affiliation patterns within the AMB, we used the clique percolation method (CPM) [32] to build a community dynamics model and to evaluate the degree to which each individual was part of an affiliation *COMMUNITY*.

The CPM builds communities from *k*-cliques, in which a *COMMUNITY* consists of those individuals engaged in close affiliation by the maximum of *k*-cliques. Communities were constructed independently from behavioural data involving proximity and grooming. We determined the weight of affiliation between two individuals (*a* and *b*) at time *t* following methods described by Palla *et al*. [33] as follows (see the electronic supplementary material for details):
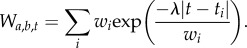


To match the GPS data for both the AMB and the BB, we built the *COMMUNITY* model beginning on 14 October 2012 for a total of 90 consecutive days, which enabled the performance of regression analysis.

#### (iii) Dominance

Data on agonistic and submissive behaviours were used to determine dominance rank among individuals in the GNG-AMB. Agnostic behaviours consisted of *biting*, *fighting*, *chasing*, *lunging*, *supplanting* and *vocal threatening*; submissive behaviours consisted of *avoidance*, *fleeing* and *crouching* (see the electronic supplementary material for definitions).

We used the normalized David's score (NDS) method to describe dominance hierarchies, which we characterized into two properties: linearity and steepness [34] (see the electronic supplementary material for details). The NDS measures dominance based on a calculation of an individual's dyadic proportions of wins combined with an unweighted and a weighted sum of its dyadic proportions of losses. We first calculate the DS for individual *i*, the baseline below normal which is given by
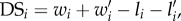
and then convert the DS into a normalized DS (NDS) by
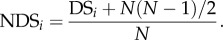


Steepness ranges from 0 to 1; a high steepness value indicates a rigid hierarchy (see the electronic supplementary material for details).

### (c) Genetic analysis

Faecal and hair samples were collected non-invasively for genetic analysis and were stored in DETs (20% DMSO, 0.25 M 106 sodium-EDTA, 100 mM Tris-HCl, pH 7.5, and NaCl to saturation) solution at −20°C or silica gel for drying, respectively. Hair DNA was extracted following methods described by Allen *et al*. [35], while faecal DNA was extracted using QIAamp DNA Stool Mini Kits (Qiagen, German). All samples were amplified at 19 tetra-nucleotide microsatellites (electronic supplementary material, table S1 and [36]) in an ABI Veriti Thermal Cycler. Alleles were segregated with an ABI PRISM 3100 Genetic Analyser, and their sizes relative to an internal size standard (ROX-labelled HD400) were determined using Genemapper v. 3.7 (Applied Biosystems). The software Micro-Checker v. 2.2.3 [37] was used to check our microsatellite data for scoring errors, allelic dropouts and null alleles.

We collected genetic samples from 92 individuals of the GNG-BB and 21 individuals from the GNG-AMB for estimating the reference allele frequency in the population. A Hardy–Weinberg equilibrium (HWE) test for each locus was performed using Genepop v. 4.3 [38]. Critical significance levels were corrected for multiple testing following the sequential Bonferroni procedure [39], and no locus significantly deviated from the HWE after Bonferroni adjustment (*α* = 0.05). The most probable cause of departure from the HWE was predicted using Micro-Checker v. 2.2.3 [37], with the potential presence of null alleles detected at loci *D10s2483* and *D10s676*. There was no evidence of large allelic dropout and scoring errors.

The relatedness coefficient between dyads of all AMB members was estimated using Lynch & Ritland's [40] estimator, with null allele corrected by PolyRelatedness v. 1.6 [41], in which the frequency of null alleles was estimated with an expectation–maximization algorithm [42].

Individuals within the AMB were clustered by the unweighted pair group method with arithmetic mean, where the relatedness between two clusters was defined as the expected estimated relatedness by randomly drawing two individuals, one each from each of the two clusters:
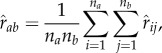
where 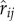 was the estimated relatedness between individuals' *i* and *j*, *a* and *b* were clusters, and *n_a_* and *n_b_* were the number of individuals within *a* and *b*, respectively. Initially, each individual defined a cluster; the two clusters that had the highest 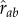 were merged repeatedly until there was only one cluster remaining.

### (d) Analysis of factors contributing to clique patterns

We combined data on kinship, dominance, age and social interactions to measure the contribution rate of each factor on clique formation and membership. Factors were separately converted into distance matrices (pairwise relatedness, NDS difference, age difference and dyadic DAIs) and correlated with the proximity HWI matrix.

Although the Mantel test [43] can measure the degree of association between two distance matrices, this method cannot simultaneously compare multiple factors. Therefore, we developed a novel higher-order partial Mantel test (HPMT) mathematical model to determine whether multiple independent matrices were significantly correlated with the dependent matrix, while estimating the contribution rate in detail for all of the other independent matrices. The HPMT originated from the Pearson's correlation coefficient:
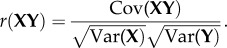


Because the social organization and affiliation patterns of primates are simultaneously affected by multiple factors, based on one of the independent matrices, we controlled the effects of the other multiple independent matrices and built a general linear model:
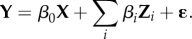


By expanding the method described by Smouse *et al*. [44], we were able to control more independent matrices (say **Z**_1_ … **Z**_k_) at the same time, and during each step we partitioned out one matrix. The *k*th-order partial correlation coefficient can be calculated from three (*k* − 1)th-order partial correlations:
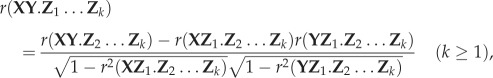
where **Z***_i_* and **Y** are independent and dependent matrices, respectively, and **X** is the independent matrix tested and is randomly permuted. The probability that 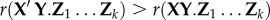 is obtained with a Monte-Carlo algorithm. Assuming that *n*_1_ out of *n*_2_ permutations has an 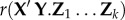 greater than 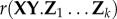, the unbiased *P* is given by 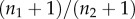. Finally, the coefficient of multiple determination is
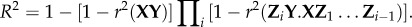


The contribution rate (CR) of a target factor is the difference between the coefficient of multiple determinations of all factors and that of other factors except the target factor. The CR value varies between 0 and 1, and the sum of all CRs cannot exceed 1 (see the electronic supplementary material for details and downloading the free novel analysis software package HPMT V1.0).

## 3. Results

### (a) Social dynamics and individual dispersal

We estimated the dynamic processes for individuals within the GNG-AMB from October 2011 to September 2016. There were 78 individuals present in the GNG-AMB, which included 34 adult males (AM), 20 sub-adult males (SAM) and 24 juvenile males (JM). We observed 39 emigrations by 32 individuals (19 AM, 6 SAM and 7 JM), 41 immigrations by 37 individuals (21 AM, 10 SAM and 6 JM), 51 inter-band transfers by 50 individuals (27 AM, 9 SAM and 14 JM) and 2 deaths (1 AM and 1 JM) (for definitions see the electronic supplementary material). Among the 51 inter-band transfers, there were 13 OMU residential male takeovers, involving males leaving the AMB and replacing existing residential males within the BB; 22 natal transfers in which a young bachelor male emigrated for the first time from its natal OMU to become a member of the bachelor group; and 16 secondary transfers from the GNG-BB to the GNG-AMB that did not involve the usurpation of the position of a resident male (8 SAM and 14 JM).

**Figure 1.**
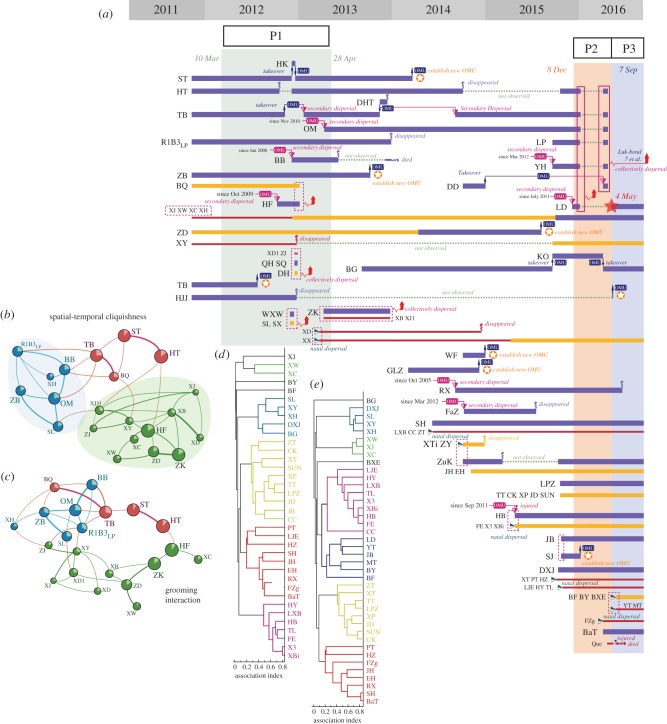
Social dynamics and individual affiliation patterns of the GNG-AMB from 2011 to 2016. (*a*) Each horizontal bar represents the social history of a single bachelor male of the GNG-AMB. The thickness and colour of each bar represents the age class of an individual bachelor male. Blue bars represent adults, orange bars sub-adults and red bars juveniles. Sociograms for proximity association (*b*) and grooming interactions (*c*) are based on social network analysis of the GNG-AMB. The width of each line connecting individuals denotes the half-weighted index (HWI) value (*b*) and the directional affiliation index (DAI) value (*c*) of a dyad. HWI values less than 0.17 (in *b*) and DAI values less than 0.19 (in *c*) indicate a weak relationship, which are not shown. (*b*) and (*c*) were built by the HWI matrix under Socprog v. 2.1 [29], and constructed by Netdraw v. 2.118 [45]. The area of each circle denotes values of the normalized David's scores of individuals and the angle of the sectors denotes the eigenvector centrality. Node colours represent different subgroups. Size fonts represent an individual's age class. (*d*) and (*e*) show the individual affiliation patterns of bachelor males within the AMB during different study periods. The clustering was performed by SNA among periods 2 and 3, respectively.

Adult males dispersed significantly more often than sub-adults and juveniles (Mann–Whitney *U* test, *U* = 344.0, *p* < 0.001). Over the course of the 5 year study, 13% of all adults dispersed more than twice. Sub-adults and juvenile males were more likely to transfer between the GNG-BB and GNG-AMB with other individuals rather than alone (figure 1*a*).

### (b) Affiliation patterns and cliquishness

During the study, behavioural data were collected in three periods (figure 1*a*) to identify the affiliation patterns and dynamics of subgroup composition of the GNG-AMB. Based on 797 h of observation from 10 March 2012 until 28 April 2013 (period 1), proximity data from 3213 scans spanning 217 days from the 21 members (8 AM, 6 SAM and 7 JM) of the GNG-AMB were collected.

**Figure 2.**
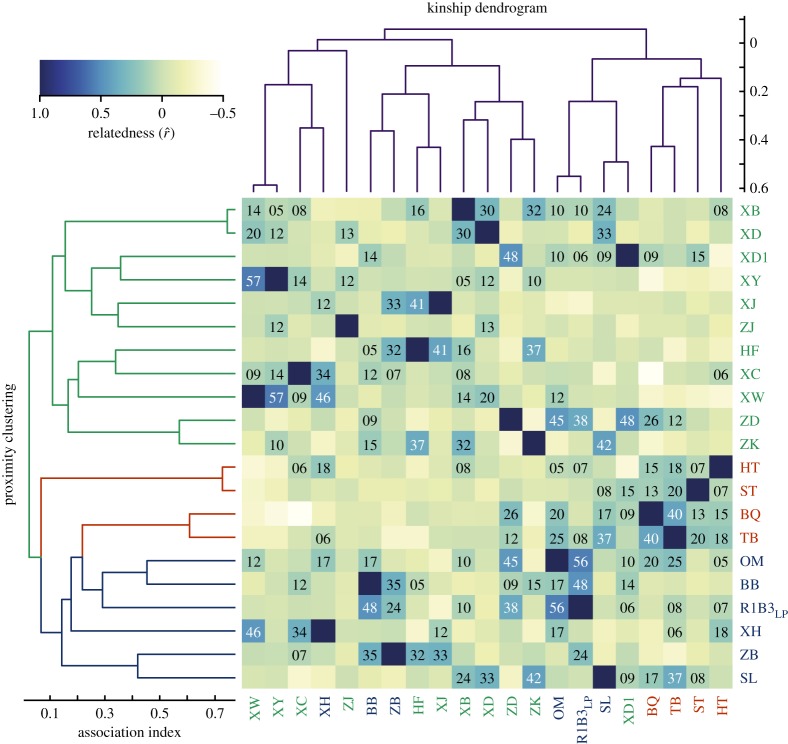
A heatmap of kinship among members of the GNG-AMB. Cell colours denote the values of the estimated relatedness coefficients. The number inside a cell represents the estimated relatedness coefficient (values > 0) and is shown as the coefficient value × 100. The two dendrograms identify the strength of proximity associations (left) and pairwise relatedness (top). Text colour (right and bottom) distinguishes members of the three AMUs obtained from the proximity data.

An HCA of SNA indicated that individuals within the AMB in period 1 are best grouped into three independent subgroups (see also figure 2; cliquishness coefficient is 0.33). The male–male HWI within the same subgroup (0.195 ± 0.018, mean ± s.e.) exhibited a stronger pattern of spatial association than males linked to different subgroups (0.050 ± 0.006) (Mann–Whitney *U* test, *U* = 1440, *p* < 0.001). We also recorded 3047 grooming bouts involving all individuals within the GNG-AMB. The average duration of each grooming bout was 186.6 ± 140.7s. SNA indicated that patterns of grooming frequency (figure 1*c*) were consistent with the association pattern identified by spatial association (figure 1*b*). The DAI for grooming between dyads of males from the same subgroup was 0.159 ± 0.017, which was significantly greater than the DAIs between members of different subgroups (0.055 ± 0.006; Mann–Whitney *U* test, *U* = 2330, *p* < 0.001). We refer to each subgroup as an all-male unit (AMU).

### (c) Correlation between kinship and behavioural affiliation

During period 1, 72 alleles from 113 individuals were segregated at 19 microsatellites. The number of alleles per locus was 3.79 ± 0.95 (mean ± s.d.). The observed heterozygosity was 0.580 ± 0.112, with the expected heterozygosity being 0.580 ± 0.095. Our results indicated a moderate level of genetic variability in the GNG-herd (electronic supplementary material, table S1). After adjusting for multiple tests, all loci in the GNG-herd were consistent with the expectations of the HWE. In addition, Wright's inbreeding coefficient was 0.000 ± 0.105, indicating a low level of inbreeding within the GNG-herd. The genetic diversity statistics of the GNG-herd are shown in the electronic supplementary material, table S1.

Based on the calculation of allele frequencies in the reference population above, the pairwise relatedness between each of the 21 bachelor males from the GNG-AMB was measured. These averaged −0.005 ± 0.013 (mean ± s.e., *n* = 210, figure 2). Both tests showed that the average values of relatedness for dyads within the same AMU was (0.042 ± 0.022, *n* = 76), significantly higher than the dyads from different AMUs (−0.032 ± 0.015, *n* = 134) (Mann–Whitney *U* test: *U* = 3998, *p* < 0.01; matrix permutation test: *p* < 0.01). Furthermore, the matrix for the dyadic affiliation index significantly correlated with the matrix for pairwise relatedness coefficients (Mantel test: *r* = 0.197, *p* < 0.001), indicating that males residing in the GNG-AMB made social affiliations based on kinship (figure 2). Such male–male bonds were thus stable and important for forming a cohesive AMU (see also figure 1*b*). Several AMUs aggregated into a modular AMB.

### (d) Dominance

During period 1, we observed 2273 instances of conflict behaviour within the GNG-AMB. These included 2194 cases of agonistic behaviours (biting 0.59%; fighting 1.91%; chasing 1.69%; lunging 9.25%; supplanting 5.97%; vocal threats 80.57%). The frequency of high-intensity aggression (e.g. biting, fighting and chasing) among bachelor males was low (chi-squared goodness of fit test: *χ*_5_^2^ = 6522.944, *p* < 0.001), which accounted for only 4.19% of all conflict behaviours. These agonistic behaviours resulted in 2092 instances of submissive behaviour (avoiding 1.00%; crouching 1.39%; fleeing 97.61%, *χ*_2_^2^ = 3889.424, *p* < 0.001).

Based on an NDS, we found a strictly linear dominance hierarchy among individuals in the AMB (steepness *K* = 0.744, *R*^2^ = 0.965; see the electronic supplementary material for NDS and rank order details). Furthermore, Mantel tests showed that dominance status was strongly and positively influenced by age (*r* = 0.813, *p* < 0.001).

### (e) Factors affecting subgroup aggregation

Based on a novel HPMT, we identified the CR of relatedness, age class and dominance status and how each factor affected subgroup composition by controlling multiple independent matrices present in the model. The results indicated that relatedness contributed most strongly to spatial proximity (dyadic HWIs) (*r* = 0.159, *p* = 0.017, CR = 0.023). However, age class (*r* = 0.105, *p* = 0.070, CR = 0.010) and dominance rank (measured using NDS) (*r* = 0.081, *p* = 0.116, CR = 0.006) explained less of the variance in the proximity matrix than did relatedness (*R*^2^ = 0.120).

We also found similar results for the effects of kinship, dominance and age on male dyadic grooming interactions (represented by DAIs) (*r* = 0.141, *p* = 0.027, CR = 0.018), age (*r* = 0.095, *p* = 0.080, CR = 0.008) and dominance (*r* = 0.094, *p* = 0.084, CR = 0.008, *R*^2^ = 0.116). Thus, kinship was also the strongest predictor of male–male social affiliations.

### (f) Male recruitment and new subgroup formation

We also monitored and recorded a specific case of a new AMU formation within the GNG-AMB. A previously reproductive male (LD) was replaced by another male during December 2015. After being forced to leave his OMU and disappearing, LD transferred to the AMB on 14 May 2016 (figure 1*a*).

**Figure 3.**
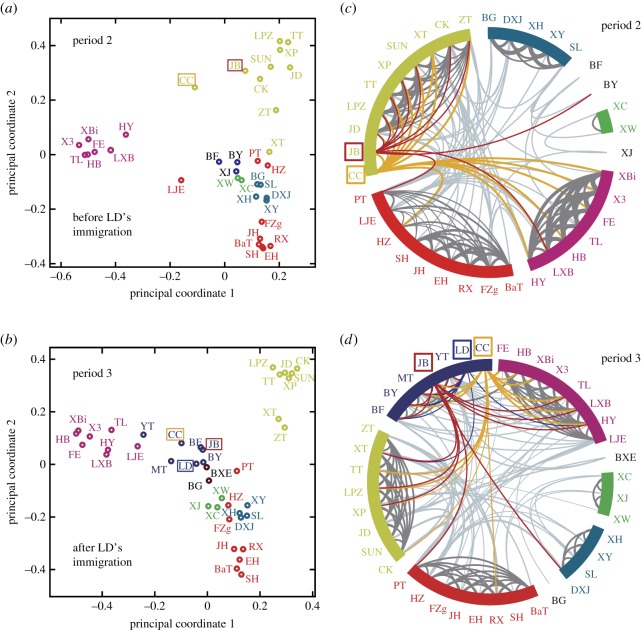
(*a,b*) Principal coordinates analysis (PCA) showing affiliation pattern shifts between individuals. Distances between nodes are inversely proportional to the square root of their HWIs and represent dyadic affiliation distances between individuals. (*c,d*) The affiliation sociograms of the GNG-AMB. The width of the bonding lines connecting individuals denotes the strength of affiliation. The filter threshold of the HWI value is 0.17. The two periods were divided by a single event involving an adult male (LD) entering the GNG-AMB and then recruiting young bachelor males already present in the AMB to form a new subgroup principally based on kinship. Node colours represent individuals belonging to different subgroups.

Based on SNA, HCA revealed five AMUs (cliquishness coefficient index (CCI) is 0.39, figure 1*d*) were present before the immigration of LD (period 2 in figure 1*a*). However, after the immigration of LD (period 3 in figure 1*a*), by recruiting young bachelor males of close kin, LD immediately associated with an adult male (JB) and several juvenile males (CC, BF, BXE and BY) to form a new AMU (CCI is 0.44, figure 1*e*). Both principal coordinates analysis and the HWI-based sociogram show the shift of affiliation patterns between the two different periods (figure 3).

Genetic analyses showed that estimated pairwise relatedness between LD and LD-AMU members was significantly higher than the average pairwise relatedness of other individuals within the GNG-AMB (Mann–Whitney *U* test: *U* = 66 068, *p* < 0.001) across periods 2 and 3.

### (g) The effects of breeding opportunities on the dynamics of male–male social affiliations

**Figure 4.**
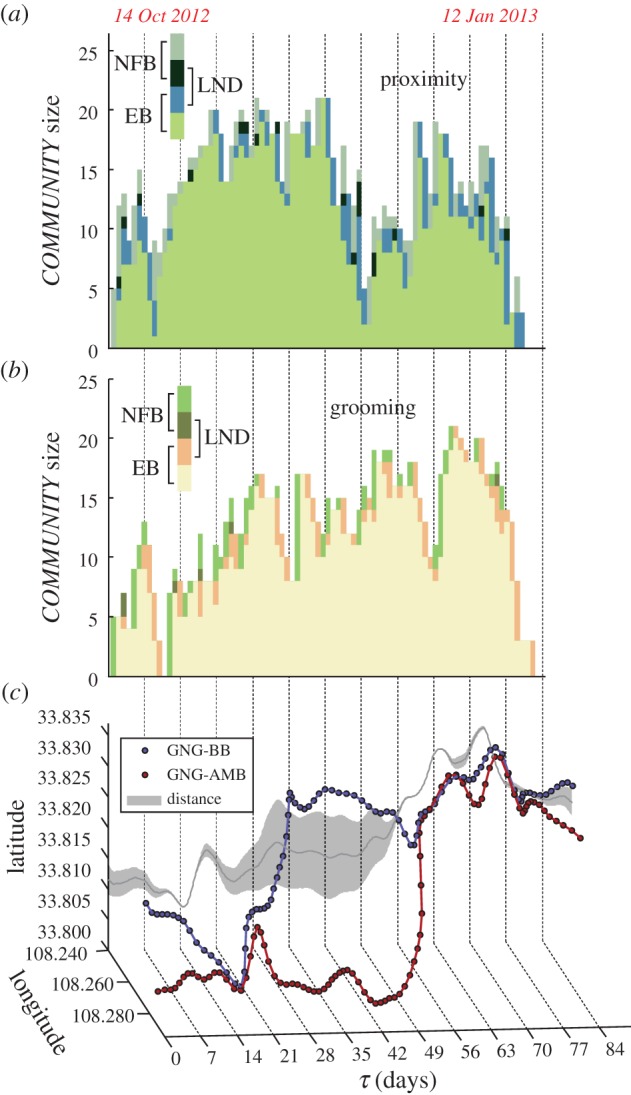
(*a,b*) The *COMMUNITY* size in each column representing the number of individuals affiliated together on a given day, which was built by the clique percolation method (CPM). EB, individuals who were members of the present *COMMUNITY* but also existed in the *COMMUNITY* of the previous day; NFB, males who joined the *COMMUNITY* on that day; and LND, males who left the *COMMUNITY* on the next day. (*c*) Dynamic distance between the GNG-BB and the GNG-AMB as estimated by satellite telemetry. The lines in this three-dimensional representation are the longitudinal and latitudinal positions of each band over time. The vertical projection shows the spatial distance between the AMB and the BB.

**Figure 5.**
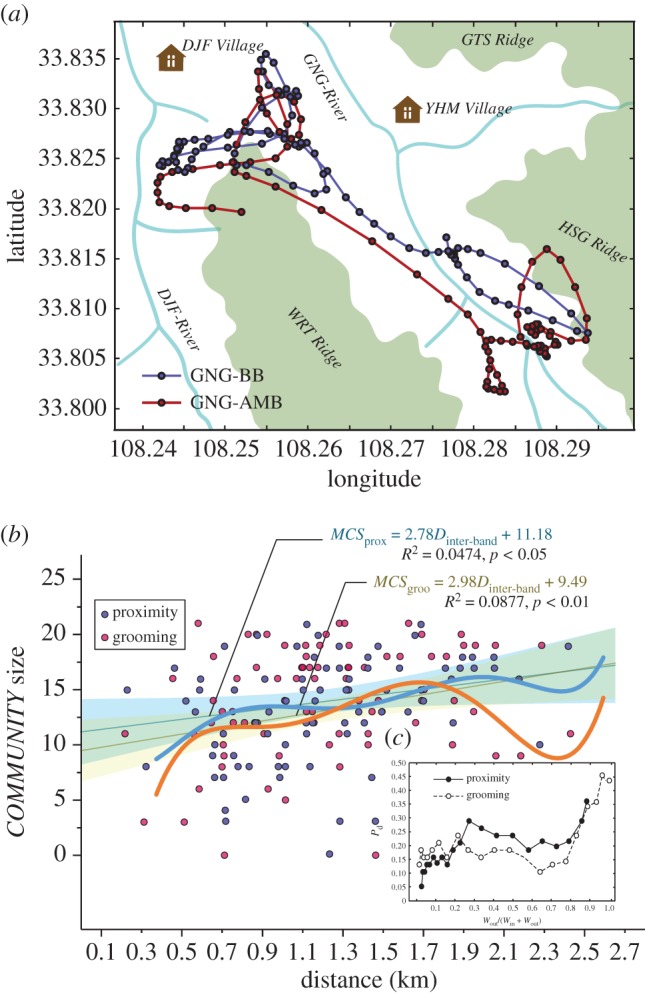
(*a*) Spatial patterns of the GNG-BB and the GNG-AMB. (*b*) The correlation between *COMMUNITY* size for each day and the distances between the BB and the AMB for each day. (*c*) The probability of an individual leaving the *COMMUNITY* as a function of the frequency of contact that individual had with other individuals outside of the *COMMUNITY*.

A *COMMUNITY* dynamics model by CPM showed how many bachelor males were engaged in close male–male bonding on a given day (figure 4*a*,*b*). The results show that *COMMUNITY* size was dynamic, and was affected by the distance between the AMB and the BB (figure 4*c*). The strength of these alliances increased as the AMB approached the BB (figure 5*a*), but was highest when the AMB was from 300 to 900 m from the BB (figure 5*b*). However, once the AMB approached within 200 m of the BB (the distance that would enable bachelor males to individually communicate with and attract females), the strength of the alliances declined steeply (figure 5*b*). This variation in bachelor male social cohesion appears to be a response to increased breeding opportunities in the presence of harem females (figure 5*c*), assuming that a female *R. roxellana* only responds to the courtship attempts of a solitary individual male.

## 4. Discussion

In many species of social mammals, adult males face the challenge of balancing intra-sexual competition and tolerance in attempting to increase individual reproductive success [46,47]. Based our study of the dynamic social interactions among male golden snub-nosed monkeys living in a multilevel society, we found that bachelor males residing in the AMB form strong and persistent affiliative bonds, based principally on kinship (figure 2). Although rank and age also represent significant factors contributing to the formation and persistence of male–male bonding, HPMT results show that kinship was the most important factor influencing male–male alliance formation. Social network analyses indicated that, within the AMB, particular sets of males formed distinct subgroups or AMUs (figure 1*b*). Closer relatedness were also shown between individuals residing in the same AMU (figure 2), as well as crucially resulting in member recruitment to form new AMUs (figure 3). Several such cohesive AMUs were thus embedded into a modular AMB alliance.

Furthermore, satellite telemetry data showed that bachelor male cohesion varied in response to social context, in particular breeding opportunities (figures 4 and 5). These interaction dynamics are consistent with a stepped strategy used by bachelor males to usurp the breeding positions of harem leader males.

Although in other mammalian MLSs males who form strong alliances are not necessary related, e.g. in Guinea baboons [3], alliances between brothers, fraternal aggregations across families and bachelor ‘clubs’ play an important role in MLSs in humans [18], bottlenose dolphins [48] and African elephants [49]. In contrast to Patzelt *et al*.'s study of Guinea baboons [3], our data suggest that kin selection may offer an explanation for male–male tolerance and social bonding in *R. roxellana*. The polygynous breeding system of snub-nosed monkeys results in high variance in male reproductive success, because only a relatively small percentage of adult males in a herd monopolize breeding opportunities. Kin-based alliances in the AMB may thus offer inclusive fitness benefits to deposed males who have lost their dominant position but assist close relatives in attaining reproductive opportunities when competition for females is high [46]. In addition, although strong kin relationships and social alliances have been reported in primate species in which males are philopatric [8], to our knowledge, this is the first evidence of a kinship-driven association network within an all-male primate social unit. Considering previous studies reporting that female snub-nosed monkeys also show kinship-based bonds and paired parallel dispersal within the BB [50], kinship-based social alliances appear to benefit individuals of both sexes in this species and play an important role in the evolution of this primate MLS.

We found that most of the individuals within each AMU were of the same age class. Our previous studies of *R. roxellana* BBs showed that juveniles (1–3.5 years of age) from different OMUs frequently play together [51,52]. Juvenile males who played together during their early development with immature males in their natal OMU or with similar aged males from other OMUs in the same BB, may thus continue to interact preferentially when they are recruited into the AMB.

Our data show that the dominance hierarchy among the males of the AMB was linearly age-based with adult bachelor males dominant over sub-adult and juvenile males and having priority of access to food resources (Mantel test: *r* = 0.813, *p* < 0.001). Although any additional short- and long-term benefits of high rank within an AMB remain unclear, given that most conflict within the AMB was mild and unrelated to reproductive competition, it is possible that recognition of rank helps individuals avoid costs associated with aggressive conflict within the AMB, as well as facilitating long-term bonding.

In *R. roxellana*, the AMB exhibits considerable home-range overlap with, and tends to follow or shadow the BB [2]. Bachelor males within the AMB actively monitor and may then approach the BB, to assess the BB males and OMU stability for opportunities to usurp the position of a BB male [26,53]. This increases the risk to breeding males of replacement by bachelor males. In this context, it has been reported that in *R. roxellana*, OMU males within the BB act collectively to defend their OMUs against rival bachelor males attempting a takeover [26]. By contrast, closely related bachelor males who form an AMU may aggregate to form an alliance and act jointly in order to enhance the possibility of approaching the BB.

Although maintaining a large AMB may potentially benefit bachelor males by facilitating successful close spatial association with the BB, these males face additional requirements in order to attract females to leave their current OMU, establish a new OMU or to usurp the monopolized position of an existing OMU male. This is because the process of OMU male replacement mostly depends on female choice for a preferred male [54]. Female *R. roxellana* only respond to the courtship attempts of a single bachelor male [55], and therefore a bachelor male has to independently attract the attention of a female and/or that female may actively solicit extra-pair copulations with an individual bachelor male when he is temporally separated from the AMB [55–57]. We found that interactions between bachelor males of the AMB became more agonistic as they approached within 200 m of the BB. At this distance, bachelor males increased their opportunities to interact with and solicit harem females. This concurs with the strength of bachelor male social affiliations steeply declining when the AMB gets close to the BB.

Our finding that members of each AMU continue to associate, fragment and then re-establish social bonds according to their distance from the BB illustrates the complicated social balance that exists between conflict and cooperation that many AMU males experience (figure 4). Our data are consistent with the hypothesis that bachelor males form alliances to help them obtain increased access to breeding opportunities against a collective of OMU breeding males. These interaction dynamics may have resulted from an ‘arms race’ associated with increased male collective action as AMBs encounter larger coalitions of OMU males defending ever-larger BBs. Male cooperation in this species thus appears to be driven by male reproductive competition, resulting in the fusion of several OMUs and AMUs characteristic of an ancestral Asian colobine to form an MLS composed of a BB and an AMB (see also the electronic supplementary material, figure S1).

Finally, the social complexity characteristic of the non-human primate MLS offers valuable insights into how the competing demands of cooperation and competition shape male–male relationships [58]. This has implications for the evolution of human social behaviour, because male–male alliances are a trademark of small- and large-scale societies and have persisted throughout much of human evolution [16,18].

## Supplementary Material

Qi et al. Methods Results Tables Figures ESM

## Supplementary Material

HPMT V1.0

## Data accessibility

The datasets supporting this article have been uploaded as part of the electronic supplementary material.
