# Supplementary material for: Male cooperation for breeding opportunities contributes to the evolution of multilevel societies
Source: Proc Biol Sci. 2017 Sep 27;284(1863):20171480. doi: 10.1098/rspb.2017.1480 (PMC5627208; doi:10.1098/rspb.2017.1480)
Supplement: Qi et al. Methods Results Tables Figures ESM [file rspb20171480supp1.docx]

**Electronic Supplementary Material**

**Male cooperation for breeding opportunities contribute to the evolution of multilevel societies**

Xiao-Guang QI, Kang HUANG, Gu FANG, Cyril C. GRUETER, Derek W. DUNN, Yu-Li LI, Weihong JI, Xiao-Yan WANG, Rong-Tao WANG, Paul A. GARBER, and Bao-Guo LI

# Methods

## Study site and subject

This study was conducted on a wild troop (West Ridge troop, WRT) of *R. roxellana* inhabiting in the Yuhuangmiao area within the Zhouzhi National Nature Reserve (ZNNR) (108º14’-108º18’E, 33º45’-33º50’N) of the Qinling Mountains, China [[1](#_ENREF_1)]. The home range of the WRT encompasses 2250 ha of mountainous forest. Vegetation in the area consists of deciduous broadleaf forest (1,380-2,200m), coniferous and deciduous broadleaf mixed forest (2,200-2,600m) and coniferous forest (2,600m and above). Depending on the time of year, lichen, bark, leaves, buds, fruits, and seeds account for the majority of golden snub-nosed monkey feeding time [[2](#_ENREF_2)].

The WRT was composed of the GNG-herd (166 individuals) and the DJF-herd (103 individuals). Each herd consisted of one breeding band formed by 7-13 one-male, multi-female reproductive units (OMUs), an all-male band (AMB), as well as solitary males moving and foraging independently within the same home range. The home range of the GNG-herd partially overlapped with the home range of the DJF-herd. During a brief three week period of the year the two herds fuse and individual’s may transfer between herds [[1](#_ENREF_1)].

Individuals were identified based on their unique physical characteristics such as patterns of pelage coloration, scars or evidence of previous injury, and the shape of fleshy nodules on both sides of the upper lip, which are present only in fully adult males. To permanently mark the individuals, an RFID identification tag (TX1411SSL) was fitted in the arm subcutaneously, and the lips of males were tattooed with a unique combination of colored spots. All individuals that appeared in the GNG-herd within observation periods were recorded and had genetic samples taken to enable identification by DNA fingerprinting.

## Behavioural analysis

### Definition of social dynamics

Dispersal of individuals within the study bachelor group included emigration, immigration and transfer [[3](#_ENREF_3), [4](#_ENREF_4)]. Emigration was defined as any bachelor male that departed from the study All-Male Band, and then not being seen again during the duration of the study. Immigration was defined as any bachelor male that entered and became established within the AMB from an unknown origin. Transfer was defined as either any male that left the AMB and replaced an existing OMU male within the breeding-band or any OMU male that had been usurped from its former OMU and had then joined the AMB.

### Definition of age classes

Adult males: Greater than 7 years of age, large body size, covered with bright golden long hair across the dorsum, and the presence of large granulomatous flanges on both sides of their upper lip.

Sub-adults: aged 4-7 years, smaller in size and with shorter golden hair and less well developed lip wart.

Juveniles: One to four years of age. Their body size is much smaller than that of adults. The color of their body hair is white to light brown.

### Affiliation and Social Network Analysis

We used Social Network Analysis (SNA) to measure patterns of individual social association within the GNG-AMB. SNA was calculated based on the Half-weighted Index (HWI) and analyzed using Socprog 2.1 [[5](#_ENREF_5)]. Affiliation behaviour was classified as an “association” to describe indirect/non-contact affiliation, and “interaction” to describe direct and instantaneous behaviour from one individual to another. We used proximity that two or more individuals shared the same space at the same time to represent associations, and grooming to represent interactions, respectively. Proximity data were collected using a 5 min interval scan sampling technique.

A Half-weighted Index (HWI) [[6](#_ENREF_6)] was used to measure the strength of social associations between individuals within the AMB:

$$\mathrm{HWI}_{ij}= X / \left[ X+Y_{ij}+0.5\left( Y_{i}+Y_{j} \right) \right].$$

Where $X$ is the number of the scan samples of individuals *i* and *j* when they were the nearest neighbor of each other; $Y_{i}$ or $Y_{j}$ is the number of scan samples of individual *i* or *j* when they were the nearest neighbor of individual *j* or *i*, but individual *j* or *i* was not the nearest neighbor for individual *i* or *j*; $Y_{ij}$ is the number of scan samples in which individuals *i* and *j* had other nearest neighbors.

Grooming data were collected using an all occurrence sampling method and the interactions between each individual within the AMB was measured by the Directional Affiliation Index (DAI), with

$$\mathrm{DAI}_{ij} = \frac{G_{ij}+G_{ji}}{\sum_{j} G_{ij}+\sum_{i} G_{ji}}.$$

Where $G_{ij}$ as the total number of times grooming was initiated by individual *i* to *j*. Sociograms to describe the patterns of individual association and interaction were constructed using Ucinet 1.0 [[7](#_ENREF_7)] software respectively. Chi-Square goodness of fit tests were used to evaluate whether agonistic and submissive behaviours were evenly distributed within partner dyads.

### Cliquishness

To estimate the cliquishness (subgroup) or evidence of social networks composed of multiple individuals, we performed a Hierarchical Clustering Analysis (HCA) of SNA. A clique was defined as an AMU within the GNG-AMB. To evaluate the stability of individual affiliation patterns and AMUs within the AMB, we used a Cliquishness Coefficient Test (CCT) of SNA and the Clique Percolation Method (CPM) [[8](#_ENREF_8)] to build a communities dynamics model and to evaluate the degree to which each individual was part of an affiliation *COMMUNITY* [[8](#_ENREF_8)].

The CPM builds communities from *k*-cliques, which are defined as a complete subgraph of *k* vertices (each pair of vertices were connected). Two *k*-cliques are considered to be adjacent if they share $k-1$ common vertices. A *COMMUNITY* is those individuals engaged in close affiliation, which is calculated by the maximal union of *k*-cliques that can be reached from each other through a series of adjacent *k*-cliques.

In our study, two kinds of communities were constructed independently from behavioural data involving proximity and grooming, with each individual represented by a vertex and the social connections between individuals represented as the edges between vertices. We assumed that the social connection between individuals began prior to our observations and lasted for some time after our observations. For simplicity, the starting date for both kinds of communities was set to Oct. 14^th^, 2012, and then continued for 90 days (as we obtained behavioural data and GPS data for both the AMB and the BB on consecutive days during this period, which could then be matched to the regression test based on distance).

Because a higher frequency of a behaviour or an extended time frame of relationships usually indicated a closer social bond, and the residual effect of an event decreases monotonously as a function of time, following Palla *et al.* [[9](#_ENREF_9)], we determine the weight of affiliation between two individuals ($a$ and $b$) at time $t$ as

$$W_{a,b,t}=\sum_{i} w_{i}\exp\left( \frac{-\lambda\left| t-t_{i} \right|}{w_{i}} \right) ,$$

where the summation runs over all considered behavioural events that occurred before $t$ in which $a$ and $b$ were involved, and $w_{i}$ denotes the weight of the *i*^th^ event occurring/ending at $t_{i}$. Proximity was sampled using a scan sampling method, and therefore the weights of all observations were equal, while the weight of grooming events was evaluated by the length of the grooming event. The direction of a grooming events was not considered. Finally, the constant $\lambda$ is an empirical coefficient. A higher value means a higher velocity of behavioural decay. The weight matrix at time *t* was converted to a binary matrix with an empirical threshold $W^{*}$.

When applied to weighted networks, the CPM method has three parameters: the *k*-clique size *k*, weight threshold $W^{*}$ and decay coefficient $\lambda$. By increasing *k* or $W^{*}$, the communities start to reduce in size and fall apart. The criterion used to choose these parameters is based on identifying a *COMMUNITY* structure that is as highly fixed structured as possible: at the highest *k* value for which a giant *COMMUNITY* may emerge, $W^{*}$ is falls just below the critical point [[9](#_ENREF_9)]. The values of these parameters in our study were $k=3$, $W^{*}= 0.005$, $\lambda=0.008$ for the grooming *COMMUNITY*, and $k=3$, $W^{*}= 0.7$, $\lambda=0.00017$ for the proximity *COMMUNITY*.

### Dominance

Data on agonistic and submissive behaviours were used to determine dominance rank among individuals in the GNG-AMB. We used a Normalized David’s Score (NDS) method to describe dominance hierarchies, which we characterized into two properties: linearity and steepness [[10](#_ENREF_10)]. Linearity has traditionally been examined using broad types of matrix methods, however this approach is limited by the use of an ordinal rank order rather than the actual value. Matrix methods can also describe steepness using quantitative operational measures especially when dyadic dominance hierarchies interval between individuals are not equal or when species are characterized by limited agonistic behaviour. Recently researchers have used a David’s score (DS) to measure dominance that is based on a calculation of an individual’s dyadic proportions of wins combined with an unweighted and a weighted sum of its dyadic proportions of losses. This method appears to be the most suitable to describe both linearity and steepness by a single precise values [[11](#_ENREF_11), [12](#_ENREF_12)].

We calculated the normalized David’s score (NDS) for the present study [[10](#_ENREF_10)]. Calculating DS for each individual in a group of *N* individuals is based on the observed numbers of dyadic wins and losses. First, we calculated the dyadic proportion of wins $P_{ij}$ calculated. It is defined as the number of times that $i$ defeats $j$ divided by the total number of interactions between $i$ and $j$. The proportion of losses $i$ in its interactions with $j$, is denoted by $P_{ji}$, equals ${1-P}_{ij}$. To avoid an undefined proportions of wins or loses when two individuals have never interacted, $P_{ij}$ and $P_{ji}$ is set to zero [[13](#_ENREF_13)], and $P_{ij}$ also is set to zero if $i=j$. The DS for individual $i$ is given by

$$\mathrm{DS}_{i}=w_{i}+w_{i}^{'}-l_{i}-l_{i}^{'}.$$

$w_{i}$ is a summation of the winning percentages of individual $i$: $w_{i}=\sum_{j} P_{ij}$, and $w_{i}^{'}$ represents a summation of $P_{ij}$ weighted by $w_{j}$: $w_{i}^{'}=\sum_{j} P_{ij}w_{j}$. Similarly, $l_{i}$ is a summation of $i$’s proportion of losses: $l_{i}=\sum_{j} P_{ji}$, and $l_{i}^{'}$ represents a weighted summation of $P_{ji}l_{i}^{'}=\sum_{j} P_{ji}l_{j}$ [[13](#_ENREF_13), [14](#_ENREF_14)].

To obtain a measurement of steepness that varies between 0 and 1, it is necessary to convert DS into a normalized DS (NDS) by

$$\mathrm{NDS}_{i}=\frac{\mathrm{DS}_{i}+N(N-1)/2}{N}.$$

Where $N(N-1)/2$ is the highest potential David’s score that can be obtained by an individual in a group of size $N$ [[10](#_ENREF_10)]. The steepness is the absolute slope of NDS as a function of rank order, and is calculated by the least-square method. Steepness ranges from 0 to 1, a high steepness indicates a rigid hierarchy; whereas a low steepness indicates a loose hierarchy.

We define agonistic behaviour as follows:

*Biting*: One individual uses its mouth and teeth to injure another individual;

*Fighting*: One individual uses its hands to hit or scratch another individual;

*Chasing*: One individual follows another individual who continues to retreat;

*Lunging*: One individual rushes at another individual, the rushed individual stops or leaves;

*Supplanting*: When one individual approaches another individual, who leaves, and the initiator assumes the spatial location of the displaced animal;

*Vocal threatening*: One individual aggressively stares at an opponent and makes a “gu-gu” vocalization.

We define submissive behaviour as follows:

*Avoidance*: One individual is threatened by another individual. The threatened individual moves away, turns to avoid looking at the threatening animal or leaves;

*Fleeing*: One individual approaches another individual, who then moves away;

*Crouching*: When one individual threatens or approaches another individual, and the approached animal adopts a sitting or bowed position, remains still, looks down, and avoids eye contact with the approaching animal.

## Genetic analysis

### Genetic sampling and genotyping

Non-invasive fecal and hair samples were collected for the genetic analysis. Fresh fecal samples were stored in DETs (20% DMSO, 0.25 M sodium-EDTA, 100 mM Tris·HCl, pH 7.5, and NaCl to saturation) solution at -20℃. Hair samples were collected by using a stick containing adhesive tape was brushed against the monkey’s fur, and stored in silica gel for drying [[15](#_ENREF_15)].

DNA was extracted from hair samples following Allen *et al.* [[16](#_ENREF_16)], while fecal DNA was extracted using QIAamp DNA Stool Mini Kits (Qiagen, German). All samples were amplified at 19 tetra-nucleotide microsatellites (table S1) in an ABI Veriti Thermal Cycler using the following protocol: 95 °C for 5 min, followed by 30 cycles (94 °C for 30s, 55-60 °C for 45s, 72 °C for 45 s), and 72 °C for 10 min. Alleles were segregated with an ABI PRISM 3100 Genetic Analyser, and their sizes relative to an internal size standard (ROX-labeled HD400) were determined using genemapper V3.7 (Applied Biosystems). To prevent genotyping errors such as false allele and allelic dropout, homozygote genotypes were confirmed by five independent replicates, with all heterozygotes observed confirmed by at least three separate reactions [[17](#_ENREF_17)].

The software micro-checker V2.2.3 [[18](#_ENREF_18)] was used to check our microsatellite data for scoring errors, allelic dropouts and null alleles.

### Estimation of kinship

We collected genetic samples from 92 individuals of the GNG-breeding band for estimating the reference allele frequency in the population (67 hair, 25 fecal samples), and 56 individuals present in the GNG-AMB (25 hair, 31 fecal samples). All individuals genotyped were thus members of the GNG-herd.

The allele frequencies of the reference population were estimated from 113 individuals (92 from the GNG-BB, and 21 from the GNG-AMB) who had resided in the MLS over the preceding seven years. A Hardy-Weinberg equilibrium (HWE) test for each locus was performed using Genepop V4.3 [[19](#_ENREF_19)]. Critical significance levels were corrected for multiple testing following the sequential Bonferroni procedure [[20](#_ENREF_20)], and no locus significantly deviated from HWE after Bonferroni adjustment (*α* = 0.05). The most probable cause of departure from HWE was predicted using micro-checker V2.2.3 [[18](#_ENREF_18)], with the potential presence of null alleles detected at loci *D10s2483* and *D10s676*. There was no evidence of large allelic dropout and scoring errors.

The Lynch & Ritland [[21](#_ENREF_21)] estimator was chosen for subsequent analyses because this test is unbiased and produces a smaller RMSE for many relationships (e.g. non-relatives). The relatedness coefficient between dyads of all AMB members was estimated using PolyRelatedness V1.6 with null allele correction [[22](#_ENREF_22)]. The frequency of null alleles was estimated with an Expectation-Maximization algorithm [[23](#_ENREF_23)] considering the negative amplification in PolyRelatedness V1.6 [[22](#_ENREF_22)].

Individuals within the AMB were clustered by calculating an estimated relatedness coefficient using the un-weighted pair-group method with the arithmetic means (UPGMA) method, where the relatedness between two clusters was defined as the expected estimated relatedness by randomly drawing two individuals, one each from these two clusters:

$$\hat{r}_{ab}=\frac{1}{n_{a}n_{b}}\sum_{i=1}^{n_{a}} \sum_{j=1}^{n_{b}} \hat{r}_{ij}.$$

Where $\hat{r}_{ij}$ was the estimated relatedness between individuals’ $i$ and $j$, $a$ and $b$ were clusters, and $n_{a}$ and $n_{b}$ were the number of individuals within $a$ and $b$, respectively. Initially, each individual defined a cluster, the two clusters that have the highest $\hat{r}_{ab}$ were merged repeatedly until there was only one cluster remaining.

We used both Mann-Whitney *U* tests and matrix permutation tests [[15](#_ENREF_15)] to compare the mean relatedness between two kinds of dyads.

## Analysis of factors contributing to clique patterns

We combined data on kinship, dominance, age and social interactions to evaluate the contribution rate of each factor affect to the clique formations and membership. Each factor was converted into distance matrices separately (pairwise relatedness, NDS difference, age difference, and dyadic DAIs) and correlated with proximity HWIs matrices.

Although Mantel test [[24](#_ENREF_24)] can measures the degree of association between two distance matrices (denoted by $\mathbf{X}$ and $\mathbf{Y}$), however, it cannot compare multiple factors at the same time. For these factors we constructed *n*-by-*n* matrices ($n$ is the number of individuals), and each pair of individuals represented an observation. These $\frac{1}{2}n\left( n-1 \right)$ observations were dependent and the degrees of freedom of these matrices are actually $n$. The multivariate linear regression could not be used.

Therefore, we developed a novel Higher-order Partial Mantel Test (HPMT) mathematics model (free software package downloading see Supporting Information), to determine whether multiple independent matrices is significantly correlated to the dependent matrix, while estimating the contribution rate in details for the other independent matrices.

The HPMT was originated from the Pearson's correlation coefficient (also the zero^th^ -order partial correlation) between $\mathbf{X}$ and $\mathbf{Y}$ is

$$r\left( \mathbf{XY} \right)=\frac{\mathrm{Cov}\left( \mathbf{XY} \right)}{\sqrt{\mathrm{Var}\left( \mathbf{X} \right)}\sqrt{\mathrm{Var}\left( \mathbf{Y} \right)}}.$$

The single-tailed probability that the Pearson's correlation coefficient between the permuted independent matrix ($\mathbf{X}'$) and $\mathbf{Y}$ is higher than the original value $r\left( \mathbf{XY} \right)$ obtained using the Monte-Carlo algorithm. However, primates social organization and affiliation patterns are simultaneously affected by multiple factors.

Based on allowed one of the independent matrices, we controlled for other of the multiple independent matrices, and built a general linear model $\mathbf{Y}=\beta_{0}\mathbf{X}+\sum_{i} \beta_{i}\mathbf{Z}_{i}+\boldsymbol{\varepsilon}$. By expanding the method described by Smouse *et al.* [[25](#_ENREF_25)], we are able to control more partial matrices (say $\mathbf{Z}_{1}\ldots\mathbf{Z}_{k}$) at the same time, and during each step we partialed out one matrix. The *k*^th^ -order partial correlation coefficient can be calculated from three ($k-1$)^th^-order partial correlations

$$r\left( \mathbf{XY}\mathbf{.}\mathbf{Z}_{1}\boldsymbol{\ldots}\mathbf{Z}_{k} \right)=\frac{r\left( \mathbf{XY}\mathbf{.}\mathbf{Z}_{2}\boldsymbol{\ldots}\mathbf{Z}_{k} \right)-r\left( \mathbf{X}\mathbf{Z}_{1}\boldsymbol{.}\mathbf{Z}_{2}\boldsymbol{\ldots}\mathbf{Z}_{k} \right)r\left( \mathbf{Y}\mathbf{Z}_{1}\boldsymbol{.}\mathbf{Z}_{2}\boldsymbol{\ldots}\mathbf{Z}_{k} \right)}{\sqrt{1-r^{2}\left( \mathbf{X}\mathbf{Z}_{1}\boldsymbol{.}\mathbf{Z}_{2}\boldsymbol{\ldots}\mathbf{Z}_{k} \right)}\sqrt{1-r^{2}\left( \mathbf{Y}\mathbf{Z}_{1}\boldsymbol{.}\mathbf{Z}_{2}\boldsymbol{\ldots}\mathbf{Z}_{k} \right)}}\left( k\geq1 \right).$$

Where $\mathbf{Z}_{i}$ and $\mathbf{Y}$ are independent and dependent matrices, respectively, and $\mathbf{X}$ is the independent matrix tested and is randomly permuted. The probability that $r\left( \mathbf{X}\mathbf{'}\mathbf{Y.}\mathbf{Z}_{1}\boldsymbol{\ldots}\mathbf{Z}_{k} \right)>r\left( \mathbf{XY.}\mathbf{Z}_{1}\boldsymbol{\ldots}\mathbf{Z}_{k} \right)$ is obtained with Monte-Carlo algorithm. Assuming $n_{1}$ out of $n_{2}$ permutations has a $r\left( \mathbf{X}\mathbf{'}\mathbf{Y}\mathbf{.}\mathbf{Z}_{1}\boldsymbol{\ldots}\mathbf{Z}_{k} \right)$ greater than $r\left( \mathbf{XY}\mathbf{.}\mathbf{Z}_{1}\boldsymbol{\ldots}\mathbf{Z}_{k} \right)$, the unbiased *P* is given by $\frac{n_{1}+1}{n_{2}+1}$. Finally, the coefficient of multiple determination is

$$R^{2}=1-\left[ 1-r^{2}\left( \mathbf{XY} \right) \right]\prod_{i} \left[ 1-r^{2}\left( \mathbf{Z}_{i}\mathbf{Y}\mathbf{.}\mathbf{XZ}_{1}\boldsymbol{\ldots}\mathbf{Z}_{i-1} \right) \right].$$

The contribution rate (CR) of a target factor is the difference between coefficient of multiple determinations of all factors, and that of other factors except the target factor. The CR value varies between 0 and 1, and the sum of all CRs cannot exceed 1.

# Supplementary Tables

Table S1: Microsatellite marker profiles, *T*_A_ denotes the annealing temperature of the primers (°C), size denotes the size of PCR products (bp), Fluo denotes the fluorescence label, *N* denotes the number of individuals types, *k* denotes the number of alleles, *H*_O_ denotes the observed heterozygosity, *H*_E_ denotes the expected heterozygosity, PIC denotes the polymorphic information content, *F*_IS_ denotes the Wright’s inbreeding coefficient, *P*-Val denotes the Hardy-Weinberg equilibrium test.

| Locus | Primer sequence (5'-3') | *T*_A_ | Size | Fluo | Array | *N* | *k* | *H*_O_ | *H*_E_ | PIC | *F*_IS_ | *P*-Val |
| --- | --- | --- | --- | --- | --- | --- | --- | --- | --- | --- | --- | --- |
| *D10s1432* | Fwd:CAGTGGACACTAAACACAATCC Rev:TAGATTATCTAAATGGTGGATTTCC | 54 | 150 | Fam | TATC | 108 | 5 | 0.546 | 0.524 | 0.467 | -0.042 | 0.576 |
| *D10s2483* | Fwd:CAGGTTTTGCAATTGCTTTC Rev:AACCATCTCCACCCACATAA | 55 | 170 | Fam | GATA | 113 | 3 | 0.522 | 0.614 | 0.535 | 0.150 | 0.170 |
| *D10s676* | Fwd:GAGAACAGACCCCCAAATCT Rev:ATTTCAGTTTTACTATGTGCATGC | 61 | 210 | Tamra | GATA | 36 | 2 | 0.333 | 0.451 | 0.346 | 0.262 | 0.142 |
| *D12s375* | Fwd:TTGTTGAGGGTCTTTCTCCA Rev:TCTTCTTATTTGGAAAAGTAACCC | 60 | 180 | Tamra | TATC | 37 | 3 | 0.568 | 0.516 | 0.392 | -0.101 | 0.606 |
| *D14s306* | Fwd:AAAGCTACATCCAAATTAGGTAGG Rev:TGACAAAGAAACTAAAATGTCCC | 52 | 190 | Tamra | GATA | 111 | 4 | 0.712 | 0.654 | 0.587 | -0.089 | 0.076 |
| *D19s248* | Fwd:GTCCAAGGAGACAGAGCCA Rev:ACTGTGCCTGACTTCTGCT | 61 | 104 | Hex | GATA | 109 | 3 | 0.706 | 0.613 | 0.538 | -0.152 | 0.127 |
| *D19s582* | Fwd:TGTGAGCAGAGAGATGGACA Rev:ACAGTGAGTTTGATCTCTAGCA | 61 | 130 | Fam | TATC | 35 | 3 | 0.429 | 0.384 | 0.346 | -0.117 | 1.000 |
| *D21s2054* | Fwd:GCAGTAAATGTCTATGAAACAAGG Rev:ATGATAGGTAGATGGATCAATTAGA | 53 | 180 | Tamra | TATC | 112 | 3 | 0.518 | 0.536 | 0.424 | 0.034 | 0.201 |
| *D3s1766* | Fwd:ACCACATGAGCCAATTCTGT Rev:ACCCAATTATGGTGTTGTTACC | 60 | 230 | Hex | TATC | 112 | 4 | 0.741 | 0.639 | 0.569 | -0.160 | 0.368 |
| *D6s1036* | Fwd:ATCCCAACTCTTAAATGGGC Rev:TTCCATGGCAGAAATTGTTT | 53 | 260 | Tamra | TATC | 107 | 5 | 0.720 | 0.765 | 0.723 | 0.059 | 0.160 |
| *D6s1040* | Fwd:GAATGCAGGACTGTTTCTGG Rev:TATATTTCTTGGGAAAGATAGATGG | 60 | 220 | Tamra | TATC | 84 | 5 | 0.607 | 0.615 | 0.535 | 0.013 | 0.835 |
| *D6s501* | Fwd:GCTGGAAACTGATAAGGGCT Rev:GCCACCCTGGCTAAGTTACT | 58 | 160 | Fam | TATC | 109 | 4 | 0.606 | 0.616 | 0.533 | 0.016 | 0.827 |
| *D7s1804* | Fwd:TTCAAGTGGTTGGGTTCACT Rev:TGGGTCTAGTCCAGTGGTGT | 60 | 240 | Hex | TATC | 43 | 5 | 0.442 | 0.456 | 0.404 | 0.031 | 0.326 |
| *D7s2204* | Fwd:TCATGACAAAACAGAAATTAAGTG Rev:AGTAAATGGAATTGCTTGTTACC | 54 | 260 | Hex | TATC | 113 | 5 | 0.708 | 0.707 | 0.657 | -0.001 | 0.420 |
| *D7s820* | Fwd:ATGTTGGTCAGGCTGACTATG Rev:GATTCCACATTTATCCTCATTGAC | 61 | 250 | Hex | GATA | 109 | 5 | 0.651 | 0.714 | 0.662 | 0.088 | 0.862 |
| *D8s1049* | Fwd:TAAGTCAAACAAGCAAAGTGC Rev:CCTCCTATTGCTTTTTCCAAA | 55 | 140 | Fam | GATA | 34 | 3 | 0.559 | 0.522 | 0.396 | -0.071 | 0.731 |
| *D9s252* | Fwd:ACCATGATTTGTCAACTCCTA Rev:ACAATGAACATCCATATACCC | 56 | 220 | Tamra | GATA | 43 | 3 | 0.442 | 0.498 | 0.442 | 0.112 | 0.378 |
| *D9s905* | Fwd:GTGGGAAAATTGGCCTAAGT Rev:CTTCTGAGCCTCACACCTGT | 54 | 280 | Hex | ATTC | 107 | 4 | 0.645 | 0.608 | 0.540 | -0.061 | 0.095 |
| *TPOX* | Fwd:GCACAGAACAGGCACTTAGG Rev:CCAAAATTGAACTCCTCA | 61 | 230 | Hex | AATG | 101 | 3 | 0.574 | 0.594 | 0.518 | 0.034 | 0.530 |

Table S2 Proximity Matrix: One minus the Half-Weight Index calculated from proximity data (in order to generate a distance matrix, where the matrix is symmetric and the diagonal elements are zeros)

|  | OM | BB | BQ | ZB | HF | R1B3_LP_ | HT | TB | SL | ST | XB | XC | XD | XD1 | XH | XJ | XW | XY | ZD | ZJ | ZK |
| --- | --- | --- | --- | --- | --- | --- | --- | --- | --- | --- | --- | --- | --- | --- | --- | --- | --- | --- | --- | --- | --- |
| OM | 0.000 | 0.548 | 0.864 | 0.634 | 1.000 | 0.791 | 1.000 | 0.727 | 0.566 | 1.000 | 0.977 | 1.000 | 1.000 | 0.978 | 0.837 | 1.000 | 1.000 | 1.000 | 1.000 | 1.000 | 0.974 |
| BB | 0.548 | 0.000 | 0.727 | 0.878 | 1.000 | 0.628 | 1.000 | 0.773 | 0.952 | 0.971 | 0.932 | 0.976 | 1.000 | 0.955 | 0.744 | 0.957 | 0.972 | 1.000 | 1.000 | 0.976 | 0.974 |
| BQ | 0.864 | 0.727 | 0.000 | 0.953 | 1.000 | 0.844 | 0.892 | 0.391 | 0.954 | 0.781 | 0.935 | 0.953 | 0.957 | 0.849 | 0.867 | 0.854 | 1.000 | 0.809 | 1.000 | 0.909 | 0.951 |
| ZB | 0.634 | 0.878 | 0.953 | 0.000 | 1.000 | 0.762 | 1.000 | 0.930 | 0.580 | 0.970 | 1.000 | 0.975 | 1.000 | 0.908 | 0.881 | 0.933 | 0.971 | 0.886 | 1.000 | 0.976 | 1.000 |
| HF | 1.000 | 1.000 | 1.000 | 1.000 | 0.000 | 1.000 | 0.811 | 1.000 | 1.000 | 1.000 | 0.803 | 0.662 | 1.000 | 0.917 | 1.000 | 0.973 | 0.852 | 0.726 | 0.818 | 0.970 | 0.705 |
| R1B3_LP_ | 0.791 | 0.628 | 0.844 | 0.762 | 1.000 | 0.000 | 0.944 | 0.756 | 0.906 | 0.972 | 0.956 | 1.000 | 0.957 | 0.890 | 0.795 | 0.915 | 1.000 | 0.870 | 0.976 | 0.884 | 1.000 |
| HT | 1.000 | 1.000 | 0.892 | 1.000 | 0.811 | 0.944 | 0.000 | 0.865 | 1.000 | 0.273 | 0.973 | 0.971 | 1.000 | 0.813 | 1.000 | 0.974 | 0.965 | 0.921 | 1.000 | 1.000 | 0.969 |
| TB | 0.727 | 0.773 | 0.391 | 0.930 | 1.000 | 0.756 | 0.865 | 0.000 | 0.908 | 0.808 | 1.000 | 1.000 | 0.979 | 0.914 | 0.867 | 0.979 | 1.000 | 0.851 | 0.954 | 1.000 | 1.000 |
| SL | 0.566 | 0.952 | 0.954 | 0.580 | 1.000 | 0.906 | 1.000 | 0.908 | 0.000 | 1.000 | 0.908 | 0.975 | 0.933 | 0.818 | 0.953 | 0.912 | 1.000 | 0.910 | 1.000 | 1.000 | 0.974 |
| ST | 1.000 | 0.971 | 0.781 | 0.970 | 1.000 | 0.972 | 0.273 | 0.808 | 1.000 | 0.000 | 0.973 | 1.000 | 1.000 | 1.000 | 0.972 | 1.000 | 1.000 | 0.947 | 1.000 | 1.000 | 1.000 |
| XB | 0.977 | 0.932 | 0.935 | 1.000 | 0.803 | 0.956 | 0.973 | 1.000 | 0.908 | 0.973 | 0.000 | 0.907 | 0.255 | 0.828 | 0.911 | 0.750 | 0.867 | 0.830 | 0.931 | 0.909 | 0.780 |
| XC | 1.000 | 0.976 | 0.953 | 0.975 | 0.662 | 1.000 | 0.971 | 1.000 | 0.975 | 1.000 | 0.907 | 0.000 | 0.864 | 0.862 | 0.952 | 0.867 | 0.739 | 0.864 | 0.852 | 0.683 | 0.974 |
| XD | 1.000 | 1.000 | 0.957 | 1.000 | 1.000 | 0.957 | 1.000 | 0.979 | 0.933 | 1.000 | 0.255 | 0.864 | 0.000 | 0.853 | 0.957 | 0.816 | 0.948 | 0.833 | 0.933 | 0.933 | 0.952 |
| XD1 | 0.978 | 0.955 | 0.849 | 0.908 | 0.917 | 0.890 | 0.813 | 0.914 | 0.818 | 1.000 | 0.828 | 0.862 | 0.853 | 0.000 | 0.846 | 0.732 | 0.895 | 0.642 | 0.886 | 0.820 | 0.880 |
| XH | 0.837 | 0.744 | 0.867 | 0.881 | 1.000 | 0.795 | 1.000 | 0.867 | 0.953 | 0.972 | 0.911 | 0.952 | 0.957 | 0.846 | 0.000 | 0.702 | 0.863 | 0.957 | 0.976 | 0.814 | 0.950 |
| XJ | 1.000 | 0.957 | 0.854 | 0.933 | 0.973 | 0.915 | 0.974 | 0.979 | 0.912 | 1.000 | 0.750 | 0.867 | 0.816 | 0.732 | 0.702 | 0.000 | 0.873 | 0.796 | 0.912 | 0.652 | 0.953 |
| XW | 1.000 | 0.972 | 1.000 | 0.971 | 0.852 | 1.000 | 0.965 | 1.000 | 1.000 | 1.000 | 0.867 | 0.739 | 0.948 | 0.895 | 0.863 | 0.873 | 0.000 | 0.844 | 0.686 | 0.887 | 0.969 |
| XY | 1.000 | 1.000 | 0.809 | 0.886 | 0.726 | 0.870 | 0.921 | 0.851 | 0.910 | 0.947 | 0.830 | 0.864 | 0.833 | 0.642 | 0.957 | 0.796 | 0.844 | 0.000 | 0.888 | 0.644 | 0.929 |
| ZD | 1.000 | 1.000 | 1.000 | 1.000 | 0.818 | 0.976 | 1.000 | 0.954 | 1.000 | 1.000 | 0.931 | 0.852 | 0.933 | 0.886 | 0.976 | 0.912 | 0.686 | 0.888 | 0.000 | 0.928 | 0.429 |
| ZJ | 1.000 | 0.976 | 0.909 | 0.976 | 0.970 | 0.884 | 1.000 | 1.000 | 1.000 | 1.000 | 0.909 | 0.683 | 0.933 | 0.820 | 0.814 | 0.652 | 0.887 | 0.644 | 0.928 | 0.000 | 1.000 |
| ZK | 0.974 | 0.974 | 0.951 | 1.000 | 0.705 | 1.000 | 0.969 | 1.000 | 0.974 | 1.000 | 0.780 | 0.974 | 0.952 | 0.880 | 0.950 | 0.953 | 0.969 | 0.929 | 0.429 | 1.000 | 0.000 |

Table S3 Grooming Matrix: One minus the Directional Affiliation Index calculated from grooming data

|  | OM | BB | BQ | ZB | HF | R1B3_LP_ | HT | TB | SL | ST | XB | XC | XD | XD1 | XH | XJ | XW | XY | ZD | ZJ | ZK |
| --- | --- | --- | --- | --- | --- | --- | --- | --- | --- | --- | --- | --- | --- | --- | --- | --- | --- | --- | --- | --- | --- |
| OM | 0.000 | 0.536 | 0.933 | 0.610 | 1.000 | 0.694 | 1.000 | 0.776 | 0.738 | 1.000 | 1.000 | 1.000 | 1.000 | 0.963 | 0.964 | 1.000 | 1.000 | 0.983 | 1.000 | 1.000 | 0.957 |
| BB | 0.536 | 0.000 | 0.764 | 0.877 | 1.000 | 0.740 | 1.000 | 0.626 | 0.891 | 1.000 | 0.968 | 0.980 | 1.000 | 0.965 | 0.846 | 0.874 | 1.000 | 0.967 | 1.000 | 0.981 | 0.960 |
| BQ | 0.933 | 0.764 | 0.000 | 0.808 | 1.000 | 0.841 | 0.902 | 0.407 | 0.935 | 0.926 | 0.933 | 0.958 | 0.949 | 0.855 | 0.821 | 0.906 | 0.974 | 0.846 | 0.981 | 0.918 | 0.937 |
| ZB | 0.610 | 0.877 | 0.808 | 0.000 | 1.000 | 0.721 | 1.000 | 1.000 | 0.449 | 1.000 | 0.967 | 0.918 | 0.917 | 0.841 | 0.878 | 0.872 | 1.000 | 0.917 | 0.981 | 1.000 | 1.000 |
| HF | 1.000 | 1.000 | 1.000 | 1.000 | 0.000 | 1.000 | 0.810 | 1.000 | 1.000 | 1.000 | 0.848 | 0.709 | 0.949 | 0.971 | 1.000 | 1.000 | 0.889 | 0.922 | 0.906 | 1.000 | 0.709 |
| R1B3_LP_ | 0.694 | 0.740 | 0.841 | 0.721 | 1.000 | 0.000 | 0.977 | 0.738 | 0.922 | 1.000 | 0.919 | 0.939 | 0.885 | 0.930 | 0.897 | 0.836 | 1.000 | 0.785 | 0.907 | 0.784 | 0.960 |
| HT | 1.000 | 1.000 | 0.902 | 1.000 | 0.810 | 0.977 | 0.000 | 0.897 | 0.976 | 0.268 | 1.000 | 0.964 | 1.000 | 0.943 | 1.000 | 0.970 | 1.000 | 0.870 | 1.000 | 1.000 | 1.000 |
| TB | 0.776 | 0.626 | 0.407 | 1.000 | 1.000 | 0.738 | 0.897 | 0.000 | 0.950 | 0.792 | 0.965 | 0.956 | 0.982 | 0.925 | 0.889 | 0.961 | 1.000 | 0.841 | 0.980 | 0.979 | 1.000 |
| SL | 0.738 | 0.891 | 0.935 | 0.449 | 1.000 | 0.922 | 0.976 | 0.950 | 0.000 | 1.000 | 0.917 | 0.938 | 0.950 | 0.750 | 0.842 | 0.907 | 1.000 | 0.899 | 0.962 | 1.000 | 0.959 |
| ST | 1.000 | 1.000 | 0.926 | 1.000 | 1.000 | 1.000 | 0.268 | 0.792 | 1.000 | 0.000 | 1.000 | 1.000 | 1.000 | 1.000 | 1.000 | 1.000 | 1.000 | 0.947 | 1.000 | 0.965 | 1.000 |
| XB | 1.000 | 0.968 | 0.933 | 0.967 | 0.848 | 0.919 | 1.000 | 0.965 | 0.917 | 1.000 | 0.000 | 0.848 | 0.391 | 0.888 | 0.908 | 0.883 | 0.945 | 0.825 | 0.802 | 0.937 | 0.804 |
| XC | 1.000 | 0.980 | 0.958 | 0.918 | 0.709 | 0.939 | 0.964 | 0.956 | 0.938 | 1.000 | 0.848 | 0.000 | 0.956 | 0.952 | 0.882 | 0.924 | 0.959 | 0.844 | 0.844 | 0.887 | 0.971 |
| XD | 1.000 | 1.000 | 0.949 | 0.917 | 0.949 | 0.885 | 1.000 | 0.982 | 0.950 | 1.000 | 0.391 | 0.956 | 0.000 | 0.887 | 0.963 | 0.824 | 0.972 | 0.805 | 0.980 | 0.936 | 0.890 |
| XD1 | 0.963 | 0.965 | 0.855 | 0.841 | 0.971 | 0.930 | 0.943 | 0.925 | 0.750 | 1.000 | 0.888 | 0.952 | 0.887 | 0.000 | 0.920 | 0.787 | 1.000 | 0.752 | 0.826 | 0.884 | 0.952 |
| XH | 0.964 | 0.846 | 0.821 | 0.878 | 1.000 | 0.897 | 1.000 | 0.889 | 0.842 | 1.000 | 0.908 | 0.882 | 0.963 | 0.920 | 0.000 | 0.896 | 0.939 | 0.944 | 0.894 | 0.795 | 0.953 |
| XJ | 1.000 | 0.874 | 0.906 | 0.872 | 1.000 | 0.836 | 0.970 | 0.961 | 0.907 | 1.000 | 0.883 | 0.924 | 0.824 | 0.787 | 0.896 | 0.000 | 0.933 | 0.683 | 0.977 | 0.951 | 0.975 |
| XW | 1.000 | 1.000 | 0.974 | 1.000 | 0.889 | 1.000 | 1.000 | 1.000 | 1.000 | 1.000 | 0.945 | 0.959 | 0.972 | 1.000 | 0.939 | 0.933 | 0.000 | 0.972 | 0.621 | 0.885 | 1.000 |
| XY | 0.983 | 0.967 | 0.846 | 0.917 | 0.922 | 0.785 | 0.870 | 0.841 | 0.899 | 0.947 | 0.825 | 0.844 | 0.805 | 0.752 | 0.944 | 0.683 | 0.972 | 0.000 | 0.919 | 0.677 | 0.933 |
| ZD | 1.000 | 1.000 | 0.981 | 0.981 | 0.906 | 0.907 | 1.000 | 0.980 | 0.962 | 1.000 | 0.802 | 0.844 | 0.980 | 0.826 | 0.894 | 0.977 | 0.621 | 0.919 | 0.000 | 0.950 | 0.610 |
| ZJ | 1.000 | 0.981 | 0.918 | 1.000 | 1.000 | 0.784 | 1.000 | 0.979 | 1.000 | 0.965 | 0.937 | 0.887 | 0.936 | 0.884 | 0.795 | 0.951 | 0.885 | 0.677 | 0.950 | 0.000 | 0.944 |
| ZK | 0.957 | 0.960 | 0.937 | 1.000 | 0.709 | 0.960 | 1.000 | 1.000 | 0.959 | 1.000 | 0.804 | 0.971 | 0.890 | 0.952 | 0.953 | 0.975 | 1.000 | 0.933 | 0.610 | 0.944 | 0.000 |

Table S4 Kinship Matrix: One minus the relatedness coefficient estimated by Lynch & Ritland (1999) estimator with null allele frequency correction by Huang et al. (2016)

|  | OM | BB | BQ | ZB | HF | R1B3_LP_ | HT | TB | SL | ST | XB | XC | XD | XD1 | XH | XJ | XW | XY | ZD | ZJ | ZK |
| --- | --- | --- | --- | --- | --- | --- | --- | --- | --- | --- | --- | --- | --- | --- | --- | --- | --- | --- | --- | --- | --- |
| OM | 0.000 | 0.834 | 0.803 | 1.085 | 1.138 | 0.441 | 0.949 | 0.754 | 0.970 | 1.080 | 0.898 | 0.983 | 1.041 | 0.902 | 0.826 | 1.249 | 0.878 | 1.051 | 0.547 | 1.030 | 1.249 |
| BB | 0.834 | 0.000 | 1.096 | 0.654 | 0.950 | 0.519 | 1.095 | 1.062 | 1.133 | 1.147 | 1.168 | 0.884 | 1.134 | 0.859 | 1.004 | 1.083 | 1.132 | 1.015 | 0.907 | 1.109 | 0.850 |
| BQ | 0.803 | 1.096 | 0.000 | 1.220 | 1.015 | 1.058 | 0.853 | 0.597 | 0.829 | 0.870 | 1.101 | 1.470 | 1.095 | 0.909 | 1.206 | 1.073 | 1.285 | 1.360 | 0.740 | 1.093 | 1.288 |
| ZB | 1.085 | 0.654 | 1.220 | 0.000 | 0.684 | 0.758 | 1.047 | 1.280 | 1.198 | 1.156 | 0.971 | 0.928 | 1.172 | 0.964 | 0.983 | 0.666 | 0.976 | 0.982 | 1.011 | 1.132 | 0.966 |
| HF | 1.138 | 0.950 | 1.015 | 0.684 | 0.000 | 1.066 | 1.233 | 1.283 | 1.001 | 1.116 | 0.836 | 1.050 | 1.071 | 1.072 | 1.283 | 0.595 | 1.064 | 1.024 | 1.251 | 1.029 | 0.632 |
| R1B3_LP_ | 0.441 | 0.519 | 1.058 | 0.758 | 1.066 | 0.000 | 0.932 | 0.920 | 1.012 | 1.016 | 0.903 | 0.994 | 1.165 | 0.941 | 1.009 | 1.289 | 1.035 | 1.054 | 0.623 | 1.120 | 1.061 |
| HT | 0.949 | 1.095 | 0.853 | 1.047 | 1.233 | 0.932 | 0.000 | 0.821 | 0.996 | 0.935 | 0.916 | 0.939 | 1.006 | 1.334 | 0.821 | 1.082 | 1.317 | 1.261 | 1.108 | 1.164 | 1.135 |
| TB | 0.754 | 1.062 | 0.597 | 1.280 | 1.283 | 0.920 | 0.821 | 0.000 | 0.627 | 0.798 | 1.007 | 1.046 | 1.008 | 0.958 | 0.942 | 1.027 | 1.227 | 1.221 | 0.877 | 1.087 | 1.195 |
| SL | 0.970 | 1.133 | 0.829 | 1.198 | 1.001 | 1.012 | 0.996 | 0.627 | 0.000 | 0.922 | 0.759 | 1.290 | 0.674 | 0.911 | 1.230 | 1.079 | 1.052 | 1.037 | 1.245 | 1.003 | 0.581 |
| ST | 1.080 | 1.147 | 0.870 | 1.156 | 1.116 | 1.016 | 0.935 | 0.798 | 0.922 | 0.000 | 1.050 | 0.995 | 1.191 | 0.855 | 1.091 | 1.146 | 1.271 | 1.202 | 0.994 | 1.035 | 1.091 |
| XB | 0.898 | 1.168 | 1.101 | 0.971 | 0.836 | 0.903 | 0.916 | 1.007 | 0.759 | 1.050 | 0.000 | 0.915 | 0.698 | 0.978 | 1.198 | 1.066 | 0.862 | 0.946 | 1.048 | 1.174 | 0.682 |
| XC | 0.983 | 0.884 | 1.470 | 0.928 | 1.050 | 0.994 | 0.939 | 1.046 | 1.290 | 0.995 | 0.915 | 0.000 | 1.032 | 1.022 | 0.658 | 0.983 | 0.910 | 0.857 | 1.137 | 1.094 | 1.009 |
| XD | 1.041 | 1.134 | 1.095 | 1.172 | 1.071 | 1.165 | 1.006 | 1.008 | 0.674 | 1.191 | 0.698 | 1.032 | 0.000 | 1.150 | 1.106 | 0.993 | 0.803 | 0.883 | 1.195 | 0.869 | 1.037 |
| XD1 | 0.902 | 0.859 | 0.909 | 0.964 | 1.072 | 0.941 | 1.334 | 0.958 | 0.911 | 0.855 | 0.978 | 1.022 | 1.150 | 0.000 | 1.118 | 1.085 | 1.016 | 1.008 | 0.516 | 1.227 | 0.993 |
| XH | 0.826 | 1.004 | 1.206 | 0.983 | 1.283 | 1.009 | 0.821 | 0.942 | 1.230 | 1.091 | 1.198 | 0.658 | 1.106 | 1.118 | 0.000 | 0.879 | 0.542 | 1.038 | 0.978 | 1.014 | 1.255 |
| XJ | 1.249 | 1.083 | 1.073 | 0.666 | 0.595 | 1.289 | 1.082 | 1.027 | 1.079 | 1.146 | 1.066 | 0.983 | 0.993 | 1.085 | 0.879 | 0.000 | 1.009 | 1.012 | 1.149 | 1.012 | 1.103 |
| XW | 0.878 | 1.132 | 1.285 | 0.976 | 1.064 | 1.035 | 1.317 | 1.227 | 1.052 | 1.271 | 0.862 | 0.910 | 0.803 | 1.016 | 0.542 | 1.009 | 0.000 | 0.428 | 1.016 | 0.997 | 1.146 |
| XY | 1.051 | 1.015 | 1.360 | 0.982 | 1.024 | 1.054 | 1.261 | 1.221 | 1.037 | 1.202 | 0.946 | 0.857 | 0.883 | 1.008 | 1.038 | 1.012 | 0.428 | 0.000 | 1.241 | 0.879 | 0.899 |
| ZD | 0.547 | 0.907 | 0.740 | 1.011 | 1.251 | 0.623 | 1.108 | 0.877 | 1.245 | 0.994 | 1.048 | 1.137 | 1.195 | 0.516 | 0.978 | 1.149 | 1.016 | 1.241 | 0.000 | 1.182 | 1.301 |
| ZJ | 1.030 | 1.109 | 1.093 | 1.132 | 1.029 | 1.120 | 1.164 | 1.087 | 1.003 | 1.035 | 1.174 | 1.094 | 0.869 | 1.227 | 1.014 | 1.012 | 0.997 | 0.879 | 1.182 | 0.000 | 1.099 |
| ZK | 1.249 | 0.850 | 1.288 | 0.966 | 0.632 | 1.061 | 1.135 | 1.195 | 0.581 | 1.091 | 0.682 | 1.009 | 1.037 | 0.993 | 1.255 | 1.103 | 1.146 | 0.899 | 1.301 | 1.099 | 0.000 |

Table S5 Dominance Matrix: Difference in Normalized David’s Score between individuals

|  | OM | BB | BQ | ZB | HF | R1B3_LP_ | HT | TB | SL | ST | XB | XC | XD | XD1 | XH | XJ | XW | XY | ZD | ZJ | ZK |
| --- | --- | --- | --- | --- | --- | --- | --- | --- | --- | --- | --- | --- | --- | --- | --- | --- | --- | --- | --- | --- | --- |
| OM | 0.000 | 2.609 | 5.590 | 4.283 | 3.907 | 3.557 | 4.265 | 1.221 | 3.970 | 1.631 | 7.868 | 7.364 | 9.295 | 6.572 | 9.187 | 10.673 | 7.933 | 8.306 | 2.843 | 8.991 | 2.199 |
| BB | 2.609 | 0.000 | 2.982 | 1.674 | 6.516 | 0.948 | 6.874 | 3.830 | 1.361 | 4.240 | 5.259 | 4.755 | 6.686 | 3.963 | 6.578 | 8.064 | 5.324 | 5.697 | 0.234 | 6.382 | 4.808 |
| BQ | 5.590 | 2.982 | 0.000 | 1.308 | 9.498 | 2.033 | 9.855 | 6.812 | 1.621 | 7.222 | 2.278 | 1.774 | 3.704 | 0.981 | 3.596 | 5.083 | 2.342 | 2.716 | 2.747 | 3.401 | 7.790 |
| ZB | 4.283 | 1.674 | 1.308 | 0.000 | 8.190 | 0.726 | 8.547 | 5.504 | 0.313 | 5.914 | 3.585 | 3.081 | 5.012 | 2.289 | 4.904 | 6.391 | 3.650 | 4.023 | 1.440 | 4.708 | 6.482 |
| HF | 3.907 | 6.516 | 9.498 | 8.190 | 0.000 | 7.464 | 0.358 | 2.686 | 7.877 | 2.276 | 11.775 | 11.271 | 13.202 | 10.479 | 13.094 | 14.580 | 11.840 | 12.213 | 6.750 | 12.898 | 1.708 |
| R1B3_LP_ | 3.557 | 0.948 | 2.033 | 0.726 | 7.464 | 0.000 | 7.822 | 4.778 | 0.413 | 5.188 | 4.311 | 3.807 | 5.738 | 3.015 | 5.630 | 7.116 | 4.376 | 4.749 | 0.714 | 5.434 | 5.756 |
| HT | 4.265 | 6.874 | 9.855 | 8.547 | 0.358 | 7.822 | 0.000 | 3.043 | 8.234 | 2.633 | 12.133 | 11.629 | 13.559 | 10.837 | 13.451 | 14.938 | 12.197 | 12.571 | 7.108 | 13.256 | 2.065 |
| TB | 1.221 | 3.830 | 6.812 | 5.504 | 2.686 | 4.778 | 3.043 | 0.000 | 5.191 | 0.410 | 9.090 | 8.585 | 10.516 | 7.793 | 10.408 | 11.895 | 9.154 | 9.527 | 4.065 | 10.213 | 0.978 |
| SL | 3.970 | 1.361 | 1.621 | 0.313 | 7.877 | 0.413 | 8.234 | 5.191 | 0.000 | 5.601 | 3.898 | 3.394 | 5.325 | 2.602 | 5.217 | 6.704 | 3.963 | 4.336 | 1.127 | 5.022 | 6.169 |
| ST | 1.631 | 4.240 | 7.222 | 5.914 | 2.276 | 5.188 | 2.633 | 0.410 | 5.601 | 0.000 | 9.500 | 8.995 | 10.926 | 8.203 | 10.818 | 12.305 | 9.564 | 9.937 | 4.474 | 10.623 | 0.568 |
| XB | 7.868 | 5.259 | 2.278 | 3.585 | 11.775 | 4.311 | 12.133 | 9.090 | 3.898 | 9.500 | 0.000 | 0.504 | 1.426 | 1.296 | 1.319 | 2.805 | 0.065 | 0.438 | 5.025 | 1.123 | 10.067 |
| XC | 7.364 | 4.755 | 1.774 | 3.081 | 11.271 | 3.807 | 11.629 | 8.585 | 3.394 | 8.995 | 0.504 | 0.000 | 1.930 | 0.792 | 1.823 | 3.309 | 0.569 | 0.942 | 4.521 | 1.627 | 9.563 |
| XD | 9.295 | 6.686 | 3.704 | 5.012 | 13.202 | 5.738 | 13.559 | 10.516 | 5.325 | 10.926 | 1.426 | 1.930 | 0.000 | 2.723 | 0.108 | 1.379 | 1.362 | 0.989 | 6.451 | 0.303 | 11.494 |
| XD1 | 6.572 | 3.963 | 0.981 | 2.289 | 10.479 | 3.015 | 10.837 | 7.793 | 2.602 | 8.203 | 1.296 | 0.792 | 2.723 | 0.000 | 2.615 | 4.101 | 1.361 | 1.734 | 3.729 | 2.419 | 8.771 |
| XH | 9.187 | 6.578 | 3.596 | 4.904 | 13.094 | 5.630 | 13.451 | 10.408 | 5.217 | 10.818 | 1.319 | 1.823 | 0.108 | 2.615 | 0.000 | 1.486 | 1.254 | 0.881 | 6.344 | 0.196 | 11.386 |
| XJ | 10.673 | 8.064 | 5.083 | 6.391 | 14.580 | 7.116 | 14.938 | 11.895 | 6.704 | 12.305 | 2.805 | 3.309 | 1.379 | 4.101 | 1.486 | 0.000 | 2.741 | 2.367 | 7.830 | 1.682 | 12.872 |
| XW | 7.933 | 5.324 | 2.342 | 3.650 | 11.840 | 4.376 | 12.197 | 9.154 | 3.963 | 9.564 | 0.065 | 0.569 | 1.362 | 1.361 | 1.254 | 2.741 | 0.000 | 0.373 | 5.090 | 1.059 | 10.132 |
| XY | 8.306 | 5.697 | 2.716 | 4.023 | 12.213 | 4.749 | 12.571 | 9.527 | 4.336 | 9.937 | 0.438 | 0.942 | 0.989 | 1.734 | 0.881 | 2.367 | 0.373 | 0.000 | 5.463 | 0.685 | 10.505 |
| ZD | 2.843 | 0.234 | 2.747 | 1.440 | 6.750 | 0.714 | 7.108 | 4.065 | 1.127 | 4.474 | 5.025 | 4.521 | 6.451 | 3.729 | 6.344 | 7.830 | 5.090 | 5.463 | 0.000 | 6.148 | 5.042 |
| ZJ | 8.991 | 6.382 | 3.401 | 4.708 | 12.898 | 5.434 | 13.256 | 10.213 | 5.022 | 10.623 | 1.123 | 1.627 | 0.303 | 2.419 | 0.196 | 1.682 | 1.059 | 0.685 | 6.148 | 0.000 | 11.190 |
| ZK | 2.199 | 4.808 | 7.790 | 6.482 | 1.708 | 5.756 | 2.065 | 0.978 | 6.169 | 0.568 | 10.067 | 9.563 | 11.494 | 8.771 | 11.386 | 12.872 | 10.132 | 10.505 | 5.042 | 11.190 | 0.000 |

Table S6 Age Matrix: Difference of ages between individuals

|  | OM | BB | BQ | ZB | HF | R1B3_LP_ | HT | TB | SL | ST | XB | XC | XD | XD1 | XH | XJ | XW | XY | ZD | ZJ | ZK |
| --- | --- | --- | --- | --- | --- | --- | --- | --- | --- | --- | --- | --- | --- | --- | --- | --- | --- | --- | --- | --- | --- |
| OM | 0.000 | 0.000 | 2.000 | 0.000 | 2.000 | 2.000 | 3.000 | 1.000 | 2.000 | 2.000 | 4.000 | 4.000 | 5.000 | 4.000 | 2.000 | 4.000 | 4.000 | 4.000 | 1.000 | 2.000 | 2.000 |
| BB | 0.000 | 0.000 | 2.000 | 0.000 | 2.000 | 2.000 | 3.000 | 1.000 | 2.000 | 2.000 | 4.000 | 4.000 | 5.000 | 4.000 | 2.000 | 4.000 | 4.000 | 4.000 | 1.000 | 2.000 | 2.000 |
| BQ | 2.000 | 2.000 | 0.000 | 2.000 | 4.000 | 0.000 | 5.000 | 3.000 | 0.000 | 4.000 | 2.000 | 2.000 | 3.000 | 2.000 | 0.000 | 2.000 | 2.000 | 2.000 | 1.000 | 0.000 | 4.000 |
| ZB | 0.000 | 0.000 | 2.000 | 0.000 | 2.000 | 2.000 | 3.000 | 1.000 | 2.000 | 2.000 | 4.000 | 4.000 | 5.000 | 4.000 | 2.000 | 4.000 | 4.000 | 4.000 | 1.000 | 2.000 | 2.000 |
| HF | 2.000 | 2.000 | 4.000 | 2.000 | 0.000 | 4.000 | 1.000 | 1.000 | 4.000 | 0.000 | 6.000 | 6.000 | 7.000 | 6.000 | 4.000 | 6.000 | 6.000 | 6.000 | 3.000 | 4.000 | 0.000 |
| R1B3_LP_ | 2.000 | 2.000 | 0.000 | 2.000 | 4.000 | 0.000 | 5.000 | 3.000 | 0.000 | 4.000 | 2.000 | 2.000 | 3.000 | 2.000 | 0.000 | 2.000 | 2.000 | 2.000 | 1.000 | 0.000 | 4.000 |
| HT | 3.000 | 3.000 | 5.000 | 3.000 | 1.000 | 5.000 | 0.000 | 2.000 | 5.000 | 1.000 | 7.000 | 7.000 | 8.000 | 7.000 | 5.000 | 7.000 | 7.000 | 7.000 | 4.000 | 5.000 | 1.000 |
| TB | 1.000 | 1.000 | 3.000 | 1.000 | 1.000 | 3.000 | 2.000 | 0.000 | 3.000 | 1.000 | 5.000 | 5.000 | 6.000 | 5.000 | 3.000 | 5.000 | 5.000 | 5.000 | 2.000 | 3.000 | 1.000 |
| SL | 2.000 | 2.000 | 0.000 | 2.000 | 4.000 | 0.000 | 5.000 | 3.000 | 0.000 | 4.000 | 2.000 | 2.000 | 3.000 | 2.000 | 0.000 | 2.000 | 2.000 | 2.000 | 1.000 | 0.000 | 4.000 |
| ST | 2.000 | 2.000 | 4.000 | 2.000 | 0.000 | 4.000 | 1.000 | 1.000 | 4.000 | 0.000 | 6.000 | 6.000 | 7.000 | 6.000 | 4.000 | 6.000 | 6.000 | 6.000 | 3.000 | 4.000 | 0.000 |
| XB | 4.000 | 4.000 | 2.000 | 4.000 | 6.000 | 2.000 | 7.000 | 5.000 | 2.000 | 6.000 | 0.000 | 0.000 | 1.000 | 0.000 | 2.000 | 0.000 | 0.000 | 0.000 | 3.000 | 2.000 | 6.000 |
| XC | 4.000 | 4.000 | 2.000 | 4.000 | 6.000 | 2.000 | 7.000 | 5.000 | 2.000 | 6.000 | 0.000 | 0.000 | 1.000 | 0.000 | 2.000 | 0.000 | 0.000 | 0.000 | 3.000 | 2.000 | 6.000 |
| XD | 5.000 | 5.000 | 3.000 | 5.000 | 7.000 | 3.000 | 8.000 | 6.000 | 3.000 | 7.000 | 1.000 | 1.000 | 0.000 | 1.000 | 3.000 | 1.000 | 1.000 | 1.000 | 4.000 | 3.000 | 7.000 |
| XD1 | 4.000 | 4.000 | 2.000 | 4.000 | 6.000 | 2.000 | 7.000 | 5.000 | 2.000 | 6.000 | 0.000 | 0.000 | 1.000 | 0.000 | 2.000 | 0.000 | 0.000 | 0.000 | 3.000 | 2.000 | 6.000 |
| XH | 2.000 | 2.000 | 0.000 | 2.000 | 4.000 | 0.000 | 5.000 | 3.000 | 0.000 | 4.000 | 2.000 | 2.000 | 3.000 | 2.000 | 0.000 | 2.000 | 2.000 | 2.000 | 1.000 | 0.000 | 4.000 |
| XJ | 4.000 | 4.000 | 2.000 | 4.000 | 6.000 | 2.000 | 7.000 | 5.000 | 2.000 | 6.000 | 0.000 | 0.000 | 1.000 | 0.000 | 2.000 | 0.000 | 0.000 | 0.000 | 3.000 | 2.000 | 6.000 |
| XW | 4.000 | 4.000 | 2.000 | 4.000 | 6.000 | 2.000 | 7.000 | 5.000 | 2.000 | 6.000 | 0.000 | 0.000 | 1.000 | 0.000 | 2.000 | 0.000 | 0.000 | 0.000 | 3.000 | 2.000 | 6.000 |
| XY | 4.000 | 4.000 | 2.000 | 4.000 | 6.000 | 2.000 | 7.000 | 5.000 | 2.000 | 6.000 | 0.000 | 0.000 | 1.000 | 0.000 | 2.000 | 0.000 | 0.000 | 0.000 | 3.000 | 2.000 | 6.000 |
| ZD | 1.000 | 1.000 | 1.000 | 1.000 | 3.000 | 1.000 | 4.000 | 2.000 | 1.000 | 3.000 | 3.000 | 3.000 | 4.000 | 3.000 | 1.000 | 3.000 | 3.000 | 3.000 | 0.000 | 1.000 | 3.000 |
| ZJ | 2.000 | 2.000 | 0.000 | 2.000 | 4.000 | 0.000 | 5.000 | 3.000 | 0.000 | 4.000 | 2.000 | 2.000 | 3.000 | 2.000 | 0.000 | 2.000 | 2.000 | 2.000 | 1.000 | 0.000 | 4.000 |
| ZK | 2.000 | 2.000 | 4.000 | 2.000 | 0.000 | 4.000 | 1.000 | 1.000 | 4.000 | 0.000 | 6.000 | 6.000 | 7.000 | 6.000 | 4.000 | 6.000 | 6.000 | 6.000 | 3.000 | 4.000 | 0.000 |

# Supplementary Figures

Figure S1. An illustration of the patterns of bachelor male affiliations in golden snub-nosed monkeys.


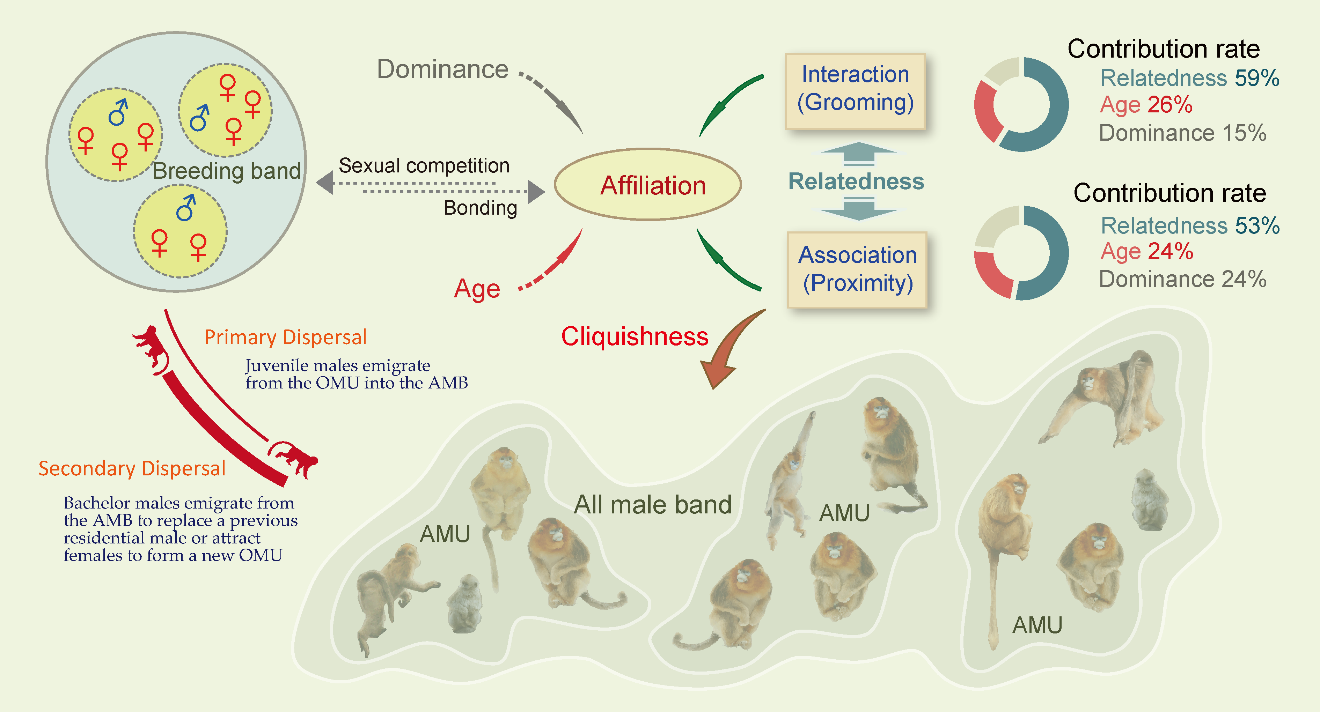


# Reference

1. Qi X.G., Garber P.A., Ji W.H., Huang Z.P., Huang K., Zhang P., Guo S.T., Wang X.W., He G., Zhang P. 2014 Satellite telemetry and social modeling offer new insights into the origin of primate multilevel societies. *Nat Commun* **5**(5), 5296.

2. Li B.G., Chen C., Ji W.H., Ren B.P. 2000 Seasonal home range changes of the Sichuan snub-nosed monkey (*Rhinopithecus roxellana*) in the Qinling Mountains of China. *Folia Primatologica* **71**(6), 375-386.

3. Moore J., Ali R. 1984 Are dispersal and inbreeding avoidance related? *Anim Behav* **32**(1), 94-112.

4. Qi X.G., Li B.G., Garber P.A., Ji W.H., Watanabe K. 2009 Social dynamics of the golden snub-nosed monkey (*Rhinopithecus roxellana*): female transfer and one-male unit succession. *Am J Primatol* **71**(8), 670-679.

5. Whitehead H. 2009 SOCPROG programs: analysing animal social structures. *Behav Ecol Sociobiol* **63**(5), 765-778.

6. Bejder L., Fletcher D., Bräger S. 1998 A method for testing association patterns of social animals. *Anim Behav* **56**(3), 719-725.

7. Borgatti S.P., Everett M.G., Freeman L.C. 2002 *UCInet for windows: software for social network analysis*. Harvard, MA, Analytic Technologies.

8. Palla G., Derényi I., Farkas I., Vicsek T. 2005 Uncovering the overlapping community structure of complex networks in nature and society. *Nature* **435**(7043), 814-818.

9. Palla G., Barabási A.L., Vicsek T. 2007 Quantifying social group evolution. *Nature* **446**(7136), 664-667.

10. de Vries H., Stevens J.M.G., Vervaecke H. 2006 Measuring and testing the steepness of dominance hierarchies. *Anim Behav* **71**(3), 585-592.

11. David H.A. 1987 Ranking from unbalanced paired-comparison data. *Biometrika* **74**(2), 432-436.

12. Gammell M.P., Han D.V., Jennings D.J., Carlin C.O.M., Hayden T.J. 2003 David's score: a more appropriate dominance ranking method than Clutton-Brock et al.'s index. *Anim Behav* **66**(3), 601-605.

13. David H.A. 1988 *The method of paired comparisons*. London, Charles Griffin.

14. de Vries H.A.N. 1998 Finding a dominance order most consistent with a linear hierarchy: a new procedure and review. *Anim Behav* **55**(4), 827-843.

15. Guo S.T., Huang K., Ji W.H., Garber P.A., Li B.G. 2015 The role of kinship in the formation of a primate multilevel society. *Am J Phys Anthropol* **156**(4), 606-613.

16. Allen M., Engström A.S., Meyers S., Handt O., Saldeen T., von Haeseler A., Paabo S., Gyllensten U. 1998 Mitochondrial DNA sequencing of shed hairs and saliva on robbery caps: sensitivity and matching probabilities. *J Forensic Sci* **43**(3), 453-466.

17. Taberlet P., Griffin S., Goossens B., Questiau S., Manceau V., Escaravage N., Waits L.P., Bouvet J. 1996 Reliable genotyping of samples with very low DNA quantities using PCR. *Nucleic Acids Res* **24**(16), 3189-3194.

18. van Oosterhout C., Hutchinson W.F., Wills D.P.M., Shipley P. 2004 MICRO-CHECKER: software for identifying and correcting genotyping errors in microsatellite data. *Mol Ecol Notes* **4**(3), 535-538.

19. Rousset F. 2008 Genepop’007: a complete re-implementation of the genepop software for Windows and Linux. *Mol Ecol Resour* **8**(1), 103-106.

20. Rice W.R. 1989 Analyzing tables of statistical tests. *Evolution* **43**(1), 223-225.

21. Lynch M., Ritland K. 1999 Estimation of pairwise relatedness with molecular markers. *Genetics* **152**(4), 1753-1766.

22. Huang K., Ritland K., Dunn D.W., Qi X.G., Guo S.T., Li B.G. 2016 Estimating relatedness in the presence of null alleles. *Genetics* **202**(1), 247-260.

23. Kalinowski S.T., Taper M.L., Marshall T.C. 2007 Revising how the computer program CERVUS accommodates genotyping error increases success in paternity assignment. *Mol Ecol* **16**(5), 1099-1106.

24. Mantel N. 1967 The detection of disease clustering and a generalized regression approach. *Cancer Res* **27**(2), 209-220.

25. Smouse P.E., Long J.C., Sokal R.R. 1986 Multiple regression and correlation extensions of the Mantel test of matrix correspondence. *Syst Zool* **35**(4), 627-632.
