# Supplementary material for: Male cooperation for breeding opportunities contributes to the evolution of multilevel societies
Source: Proc Biol Sci. 2017 Sep 27;284(1863):20171480. doi: 10.1098/rspb.2017.1480 (PMC5627208; doi:10.1098/rspb.2017.1480)
Supplement: HPMT V1.0 [file rspb20171480supp2.doc]

# HPMT v1.0

Double click the following icon to launch HPMT v1.0.

require .NET framework 2.0 runtime, support Windows platform.

# How to cite

Qi XG, Huang K, Fang G, Grueter CC, Dunn DW, Li YL, Ji WH, Wang XY, Wang RT, Garber PA, Li BG (2017) Male cooperation for breeding opportunities contribute to the evolution of multilevel societies. Proceedings of the Royal Society B, DOI.

# User Manual

In ecology, dyadic index (e.g. proximity, association, relatedness, geographic distance, genetic distance) are calculated and we usually want to test the correlation between two matrices. The dyadic index can be arranged into matrices, and each element in the upper/lower triangular matrix representing a dyad. However, the elements within a matrix are not independent. For example, for geographic distance, three individuals forms a triangular and the sum of two edges was greater than the third. Such that the ordinary linear regression cannot be used. Mantel test is used to analysis the correlation between two matrices, and partial Mantel test is able to control one matrix, and analysis the partial correlation between other two matrices. Sometimes, there are more matrices we want to control, so we develop the higher-order partial Mantel test.

This program perform a higher order partial Mantel test, which is able to control more matrices (up to five matrices, says Z1, Z2, ..., Z5), and test the correlation between other two matrices (says X and Y). The independent matrix X is permuted and we calculate the probability that the partial correlation coefficient between X and Y with Z1 to Z5 controlled after permutation is greater than the original value. A Monte-Carlo algorithm is used to obtain the probability, and it is used as a singled-tailed significance.

# Usage

Arrange the matrices in the left textbox in order X,Y,Z1,Z2,Z3,Z4,Z5.

The column and row header shows the individual, and each cells is a dyadic value in distance matrix. The rows are separated with line breaks, and the columns are separated with tabs. The example matrix is shown as follow:

Genetic pop1 pop2 pop3 pop4

pop1 0.00 0.28 0.17 0.21

pop2 0.28 0.00 0.23 0.35

pop3 0.17 0.23 0.00 0.24

pop4 0.21 0.35 0.24 0.00

The number of permutation and the decimal places can be configurated in the toolbox, then clicking the 'Permute' button. The results will shown in the right textbox. A larger number of permutation will yield a more precise significance.
